# Symmetry group factorization reveals the structure-function relation in the neural connectome of *Caenorhabditis elegans*

**DOI:** 10.1038/s41467-019-12675-8

**Published:** 2019-10-31

**Authors:** Flaviano Morone, Hernán A. Makse

**Affiliations:** Levich Institute and Physics Department, City College of New York, New York, NY 10031 USA

**Keywords:** Caenorhabditis elegans, Network models, Statistical physics, thermodynamics and nonlinear dynamics, Complex networks

## Abstract

The neural connectome of the nematode *Caenorhabditis elegans* has been completely mapped, yet in spite of being one of the smallest connectomes (302 neurons), the design principles that explain how the connectome structure determines its function remain unknown. Here, we find symmetries in the locomotion neural circuit of *C. elegans*, each characterized by its own symmetry group which can be factorized into the direct product of normal subgroups. The action of these normal subgroups partitions the connectome into sectors of neurons that match broad functional categories. Furthermore, symmetry principles predict the existence of novel finer structures inside these normal subgroups forming feedforward and recurrent networks made of blocks of imprimitivity. These blocks constitute structures made of circulant matrices nested in a hierarchy of block-circulant matrices, whose functionality is understood in terms of neural processing filters responsible for fast processing of information.

## Abstract (web summary)

The 302-neuron connectome of the nematode C. elegans has been completely mapped, yet the design principles that explain how the connectome structure determines its function are unknown. Here, the authors show that physical principles of symmetry and mathematical tools of symmetry groups can be used to understand C. elegans neural locomotion circuits.

## Introduction

There is growing consensus in present day complexity science that functions of living networked systems are controlled by the structure of interconnections between the network components^1–3^. Under this assumption, the problem of understanding how function emerges from structure^4^ can be cast in terms of the network structure itself, and this problem is, fundamentally, of a theoretical nature. Here we address this problem by considering the connectome of the neural system of the nematode *C. elegans*, a prototypical model connectome displaying complex behavior^5–11^.

**Fig. 1:**
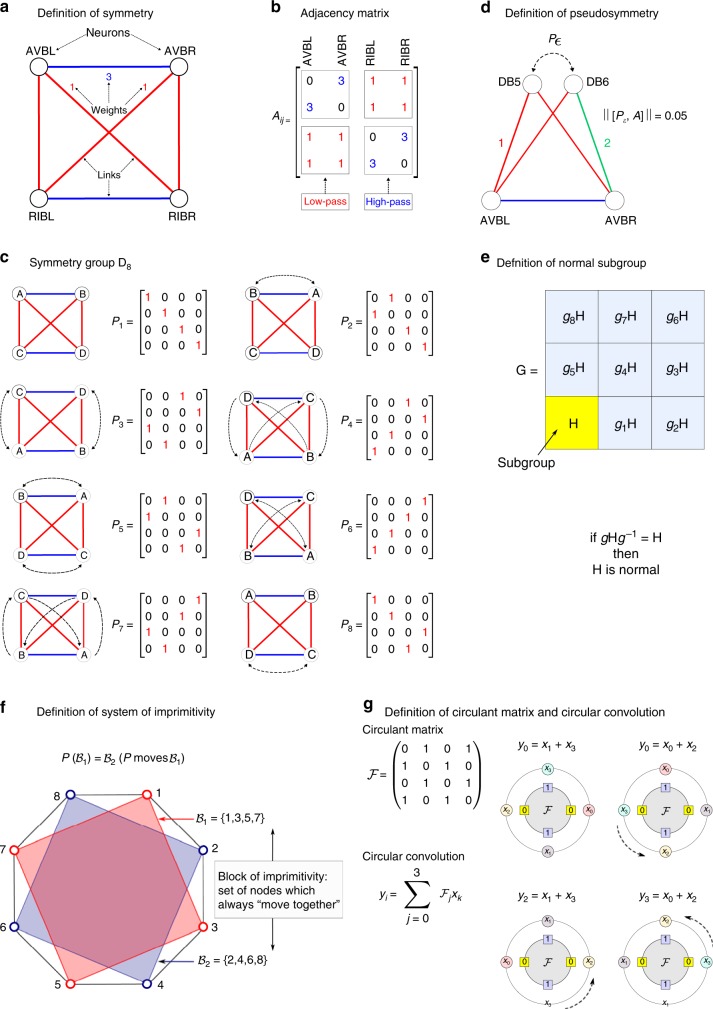
Group theoretical definitions: automorphism, symmetry groups, pseudosymmetries, normal subgroups, and blocks of imprimitivity. **a** Circuit made of gap-junction and only interneurons in the forward locomotion used to define an automorphism. These are permutation symmetries that leave the adjacency structure invariant. These symmetries then convert to a system of imprimitivity when we integrate the circuit into the full locomotion connectome. Nodes represent neurons and weighted links represent the number of gap-junctions connections between neurons from ref. ^9^. **b** Adjacency matrix of the circuit in **a**. This matrix is composed of circulant matrices: a high-pass filter ${\mathcal{H}}={\rm{circ}}(0,1)$ in the diagonal and an off-diagonal low-pass filter ${\mathcal{L}}={\mathrm{circ}}(1,1)$. The full $4\times 4$ matrix forms a block-circulant matrix ${\mathcal{B}}C={\rm{bcirc}}({\mathcal{H}},{\mathcal{L}})$^13^ (see Methods Section for definitions). **c** Symmetry group of the circuit shown in **a**, called dihedral group ${{\bf{D}}}_{{\bf{8}}}$, comprises 8 automorphisms out of the 4! = 24 possible permutations of neurons. We show each permutation matrix $P$ of each automorphism. **d** Pseudosymmetries capture inherent variabilities in the connectome from animal to animal. An example pseudosymmetry is shown ${P}_{\varepsilon }=$ DB5 $\leftrightarrow$ DB6 that breaks one link to AVBR over 18 total weighted links, giving $\varepsilon =1/18=5.5 \%$. **e** Definition of normal subgroup. A subgroup ${\bf{H}}$ is said to be normal in a group ${\bf{G}}$ if and only if ${\bf{H}}$ commutes with every element $g\in {\bf{G}}$, that is: $[g,{\bf{H}}]=g{\bf{H}}-{\bf{H}}g=0$ (see Supplementary Note 4 for a detailed explanation). **f** Definition of blocks of imprimitivity and system of imprimitivity. Simply put, a set of nodes is called a block (of imprimitivity) if all nodes in this set always ‘move together’ under any automorphism of the symmetry group. A set of blocks with such a property is thus called a system of imprimitivity (see Supplementary Note 7 for a formal definition). **g** Definition of circulant matrix and circular convolution. Matrix ${\mathcal{F}}$ appears in the forward gap-junction locomotion circuit and is called a circulant matrix. This matrix has a peculiar pattern where each row is a shift to the right by one entry of the previous row. Multiplication of ${\mathcal{F}}$ by a vector ${\bf{x}}$ gives rise to a famous operation called a circular convolution, which is used in many applications, ranging from digital signal processing, image compression, and cryptography to number theory, theoretical physics and engineering, often in connection with discrete and fast Fourier transforms, as explained in Supplementary Note 8

Specifically, we show that the building blocks of the locomotion part of the connectome are mathematically defined via its ‘*symmetry group*’^12^. The implications of this result are two-fold. First, we show that the symmetry group of locomotion circuits can be broken down into a unique factorization as the *direct product* of smaller ‘*normal subgroups*’^12^. These normal subgroups directly determine the separation of neurons into sectors. The biological significance of these sectors of neurons is measured by the fact that these sectors match known functional categories of the connectome. Second, we show that the sectors of neurons defined by each normal subgroup of the connectome can be further decomposed into ‘*blocks of imprimitivity*’^12^ made of ‘*circulant*’ matrices^13^. These circulant matrices are processing units encoding for fast signal filtering and oscillations in the locomotion function. Figure 1a–g defines the group theoretical concepts of permutation symmetry, normal subgroup, block of imprimitivity and circulant matrices needed to understand the theoretical basis of the structure-function relation in the connectome that we present here.

Our fundamental result is that symmetries of neural networks have a direct biological meaning, which can be rigorously justified using the mathematical formalism of symmetry groups. This formalism makes possible to understand the significance of the structure-function relationship: the origin of the locomotion function in *C*. elegans is connected with the existence of symmetries which uniquely assign neurons to functional categories defined by the mechanism of factorization of the symmetry group. Therefore, the structure-function relationship theoretically follows from a symmetry principle. Although the specific form of the symmetry group is different in different functions, the basic ideas and methods of our formalism are the same and can be tested for any system. The symmetry group of a network has also a strong impact on the dynamics of the system. That is, it determines the synchronization of neurons belonging to the same orbit defined by the symmetry group^14,15^. Furthermore, the form of synchrony determined by the symmetries of the connectivity structure is largely independent of the specific details of the neural dynamics. Seen globally, network symmetries may help to reveal the general principles underlying the mechanism of neural coding engraved in the connectome.

## Results

### Neural connectome: symmetry groups

The neuronal network of the hermaphrodite *C. elegans* contains 302 neurons, which are individually identifiable, and the wiring diagram includes 890 gap junctions and 6393 chemical synapses^9^. The number of neurons across animals is very consistent ^5,9^, while the gap-junctions and chemical synapses are reproducible within 25% variability from animal to animal^5,9,16,17^. Due to its small size and relative completeness, the neural network of *C*. elegans has been a formidable model system to search for design principles underpinning the structural organization and functionality of neural networks^5–11^.

We examine the forward and backward locomotion functions in *C*. elegans which have been well-characterized in the literature^5–7,10,11,18–20^. The locomotion is supported by two main functional classes of neurons called (1) command interneurons and (2) motor neurons (in addition to sensory neurons which are not studied here). The backward locomotion of the animal is supported by the activation of interneurons AVA, AVE, and AVD^18^ and AIB and RIM^10^, and motor neuron classes VA and DA. Similarly, forward locomotion is supported by the activation of motor neuron classes VB and DB through the interneuron classes AVB and PVC^5,6,10,18,19^ and RIB^10^. We use the most up-to-date connectome of gap-junctions and chemical synapses from ref. ^9^ to construct the neural circuits of forward and backward locomotion (details in Supplementary Note 1). We represent the synaptic connectivity structure by the weighted adjacency matrix ${A}_{{\mathrm{ij}}}\,\ne\,0$ if neurons $i$ connects to $j$, and ${A}_{{\mathrm{ij}}}=0$ otherwise. Gap-junctions are undirected links, ${A}_{{\mathrm{ij}}}={A}_{{\mathrm{ji}}}$, and chemical synapses are directed.

To explain the concept of symmetries and the procedure for finding the symmetry group, we first consider the circuit comprising only the interneurons connected via gap-junctions involved in the forward task (Fig. 1a, adjacency matrix in Fig. 1b, weights on the links represent the number of connections provided in ref. ^9^). Later, we will see how this circuit is integrated in the full connectome.

This sub-circuit contains $4!=24$ possible permutations of its 4 neurons. Out of these 24, only 8 are permutation symmetries as shown in Fig. 1c. A permutation symmetry, or automorphism^12,15,21,22^, is a transformation defined as a permutation of neurons which preserves the connectivity structure $A$ (see Supplementary Note 2 for detailed definition). This means that before and after the application of an automorphism, the neurons are connected exactly to the same neurons. Mathematically, if $P$ is an automorphism, then the permuted adjacency matrix $PA{P}^{-1}$ is equal to the original one, $PA{P}^{-1}=A$, or, equivalently, $P$ and $A$ commute with each other:

1 $$[P,A]\equiv PA-AP=0\ \ \iff \ \ P{\,\,}{\rm{is}}\ {\rm{a}}\ {\rm{symmetry}}.$$

For instance, the permutation ABVL $\leftrightarrow$ RIBR and ABVR $\leftrightarrow$ RIBL represented by ${P}_{6}$ in Fig. 1c is an automorphism since it leaves the connectivity intact. The set of automorphisms forms the symmetry group of the circuit, which, in this case, is the dihedral group ${{\bf{D}}}_{{\bf{8}}}$, which is the group of symmetries of a square^12^. To be called a group of transformations, the transformations need to satisfy four axioms: (1) the existence of an inverse in the group, (2) the existence of an identity, (3) the associative law, and (4) the composition law. In addition, if the transformations are commutative, then the group is called abelian.

### Pseudosymmetries

The study of the full locomotion circuit requires a generalization of the notion of network symmetry, which we call ‘pseudosymmetry’. The concept of pseudosymmetry arises naturally from the observation that connectomes vary from animal to animal, so no two worms will ever have the same connectome^5,9,16,17^. This variation is estimated experimentally to be $25 \%$ of the total connections from worm to worm, as reported in^9^ using data from^5,16,17^. We consider this variability across individual connectomes as an intrinsic property consistent with biological diversity and evolution. Furthermore, the number of connections is subject to change from animal to animal through plasticity, learning and memory^23^, so it cannot be ignored. On the other hand, while connectomes vary from animal to animal, functions developed from them, such as forward and backward locomotion, are barely distinguishable across different worms, and still show some vestige of an ideal symmetry. In fact, the locomotion function is preserved despite the 25% variation in the connectomes. Consequently, we expect that deviations from exact symmetries to be relatively ‘small’. Exact symmetries of the connectome should be considered as an idealization, and we do not expect them to be realized exactly.

Therefore, we consider pseudosymmetries of the connectome rather than perfect symmetries. Unlike perfect symmetries, defined by the commutator Eq. (1) and shown in the circuit of Fig. 1a, the definition of pseudosymmetry depends on an additional parameter, a small number $\varepsilon\,> \,0$. This parameter quantifies the uncertainty in the connectivity structure of the connectome due to natural variations across animals, and, thus, we call it the ‘*uncertainty constant*’ of the connectome. A pseudosymmetry is an approximate automorphism ${P}_{\varepsilon }$, in the sense that the commutator Eq. (1) is replaced by a non-zero but small $\varepsilon$-norm (detailed definition in Supplementary Note 3):

2 $$| | [{P}_{\varepsilon},A]| | {\,} < {\,} \varepsilon M \iff {P}_{\varepsilon }{\,\,}{\mathrm{is}}\ {\mathrm{a}}\ {\mathrm{pseudosymmetry}},$$

where $M$ is the total number of network links including weights. ${P}_{\varepsilon }$ approximates an exact symmetry in the ideal limit $\varepsilon \to 0$. The norm of the commutator, denoted as $| | [{P}_{\varepsilon },A]| |$, measures the number of links where ${P}_{\varepsilon }$ and $A$ fail to commute given an upper limit tolerance $\varepsilon$ in the fraction of links for the failure of commutativity (a simple pseudosymmetry is exemplified in Fig. 1d). The norm of the commutator in Eq. (2) is defined as the ${L}_{1}$ norm, denoted as $| | [P,A]| |$, and given by the following equation:

3 $$| | [P,A]| | =| | PA-AP| | =| | A-PA{P}^{-1}| | =\sum _{{\mathrm{ij}}}| {A}_{{\mathrm{ij}}}-{A}_{P({\mathrm{i}})P({\mathrm{j}})}| ,$$

where the last equality follows from the fact that $P$ is an isometry (i.e., $| | A| | =| | PA{P}^{-1}| |$ for any matrix $A$). We see that this definition of pseudosymmetry via a commutator resembles the uncertainty principle in quantum mechanics and, thus, perfect symmetries correspond to the ‘classical limit’ of the pseudosymmetries.

Equation (2) means that a pseudosymmetry must preserve at least a fraction $(1-\varepsilon )$ of network links. The set of pseudosymmetries of the connectome contains not only the symmetry group of the connectome $(\varepsilon =0)$ but it is augmented by the permutations that are ‘almost’ automorphisms. We note that the set of pseudosymmetries does not form a group by itself, since the pseudosymmetries do not satisfy the composition law. For instance, two pseudosymmetries (which by definition are below the threshold $\varepsilon$) may be composed into a third pseudosymmetry that breaks more than a fraction of $\varepsilon$ contacts, and, thus, does not belong to the original set of pseudosymmetries, violating composition.

Knowledge of the pseudosymmetries is particularly useful for understanding the robustness of functions under small perturbations of the connectome. This property makes it analogous to the concept of pseudospectrum which tells how much the spectrum of eigenvalues of a matrix moves respect to small perturbations, see ref. ^24^. Simply put, if the set of pseudosymmetries is clustered around the ideal symmetry group (i.e., the uncertainty constant is small), network functions are robust under small perturbations. Conversely, if it is widely spread, then functions are more likely to be lost under small perturbations. Having all concepts at hand, we move to discuss the whole forward and backward circuits and their symmetries, which, hereafter, are meant to be pseudosymmetries, although we keep using the shorthand ‘symmetry’ for lexical convenience.

**Fig. 2:**
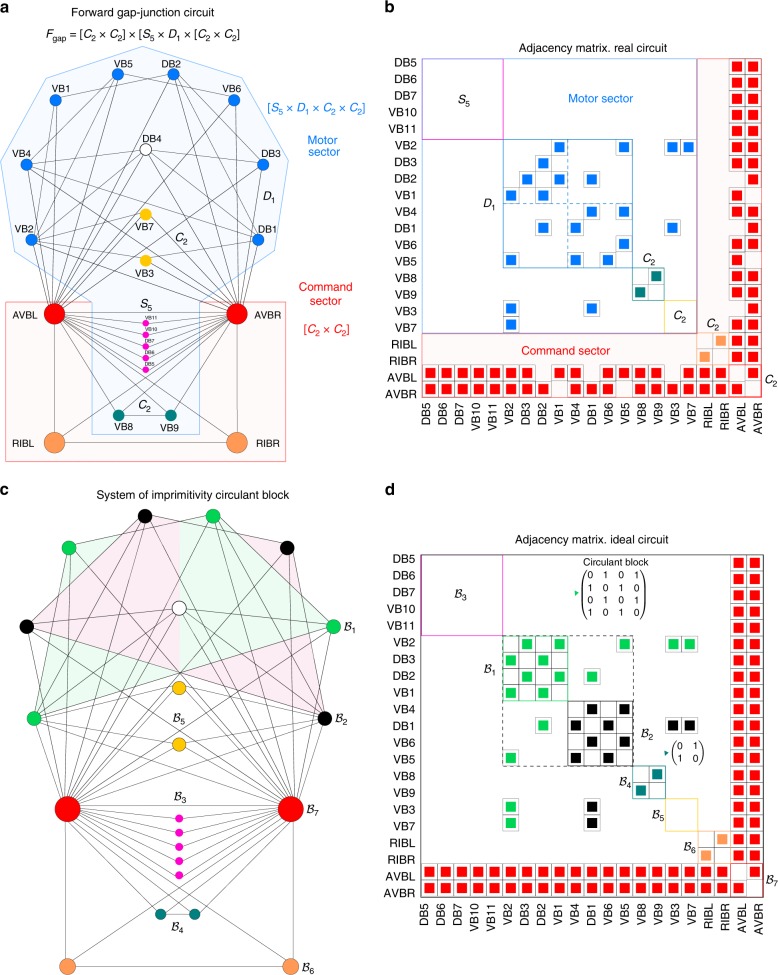
Symmetry group $${{\bf{F}}}_{{\bf{gap}}}$$ of the forward gap-junction circuit. **a** Circuit from ref. ^9^. Pseudosymmetries $${P}_{\varepsilon }$$ act on distinct sectors of neurons indicated by different colors that lead to direct product factorization of the symmetry group into normal subgroups. The normal subgroups sectors of neurons match the broad classification of command interneurons and motor neurons from the Wormatlas^25^. **b** Adjacency matrix of (**a**) showing the normal subgroup structure and its matching with broad neuronal classes. **c** Idealization of the circuit obtained from **a** by $$\varepsilon \to 0$$ leading to perfect symmetries (see Supplementary Note 3). We highlight the two 4-cycles across $${{\mathcal{B}}}_{1}$$: VB2 $$\to$$ DB3 $$\to$$ DB2 $$\to$$ VB1 $$\to$$ VB2 and its conjugate $${{\mathcal{B}}}_{2}$$: DB1 $$\to$$ VB4 $$\to$$ VB5 $$\to$$ VB6 $$\to$$ VB4 that give rise to the circulant matrix structure highlighted in the checker-board pattern in **d** of both imprimitive blocks. **d** Adjacency matrix of the ideal circuit in **c**. We highlight the two imprimitive blocks $${{\mathcal{B}}}_{1}=$$ (VB2, DB3, DB2, VB1) and $${{\mathcal{B}}}_{2}=$$ (DB1, VB4, VB5, VB6) mentioned in the text and its circulant structure in the normal subgroup $${{\bf{D}}}_{{\bf{1}}}$$. The other normal subgroups are also described by circulant blocks and correspond to imprimitive blocks: $${{\mathcal{B}}}_{3},$$$${{\mathcal{B}}}_{4},$$$${{\mathcal{B}}}_{5},$$$${{\mathcal{B}}}_{6},$$$${{\mathcal{B}}}_{7}$$, as indicated. Some of these structures also form block-circulant matrices. Each block of the adjacency matrix $$A$$ performs a fundamental signal processing task

The forward gap-junction circuit is shown in Fig. 2. This circuit has permutation symmetries, denoted as ${{\bf{F}}}_{{\bf{gap}}}$, most of which can be spotted by eye in the layout displayed in the figure. Figure 2a, b display the real circuit, the adjacency matrix and its pseudosymmetries (details of calculations in Supplementary Note 3). The uncertainty constants $\varepsilon$ of these pseudosymmetries are listed in Table 1 and fall below the upper experimental limit of 25%. Thus, all pseudosymmetries have biological significance. Figure 2c, d show an ideal circuit obtained by setting $\varepsilon =0$ compatible with the found pseudosymmetries (see Supplementary Note 3 for details on how to obtain the ideal symmetric circuit).

**Table 1:** Pseudosymmetries of the locomotion circuit

	$\varepsilon$ (%)	Subgroup	*p*-value
*Pseudosymmetry-Forward* *gap-junction*			
(RIBL, RIBR)	0.0%	${{\bf{C}}}_{{\bf{2}}}$	0.001
(VB3, VB7)	5.3%	${{\bf{C}}}_{{\bf{2}}}$	0.02
(VB8, VB9)	9.6 %	${{\bf{C}}}_{{\bf{2}}}$	0.004
(AVBL, AVBR)	24.5%	${{\bf{C}}}_{{\bf{2}}}$	0.0007
(DB5, DB6, DB7, VB10, VB11)	5.5 %	${{\bf{S}}}_{{\bf{5}}}$	0.0002
(DB1, VB2, DB2, VB5, DB3, VB4, VB1, VB6)	23.4%	${{\bf{D}}}_{{\bf{1}}}$	0.00001
*Pseudosymmetry-Backward* *gap-junction*			
(AIBL, AIBR, RIML, RIMR)	1.5%	${{\bf{D}}}_{{\bf{1}}}$	0.00001
(DA8, DA9, DA2, VA1, DA1, DA4)	6.9%	${{\bf{D}}}_{{\bf{6}}}$	$< 1{0}^{-6}$
(AVEL, AVER)	1.5%	${{\bf{C}}}_{{\bf{2}}}$	0.005
(VA4, VA5)	3.8%	${{\bf{C}}}_{{\bf{2}}}$	0.005
(VA2, VA3, VA6, VA7, VA8, VA9, VA10, VA11, VA12, DA3, DA6, DA7)	13.8%	${{\bf{S}}}_{{\bf{12}}}$	$< 1{0}^{-6}$
*Pseudosymmetry-Forward* *chemical* *synapse*			
(VB3, VB4, VB5, VB10, VB11, DB2, DB4, DB6, DB7, DB8)	3.8%	${{\bf{S}}}_{{\bf{10}}}$	0.014
(VB6, VB7, VB8, VB9)	3.8%	${{\bf{D}}}_{{\bf{1}}}$	0.0012
(PVCL, PVCR)	3.8%	${{\bf{C}}}_{{\bf{2}}}$	0.0006
(AVBL, AVBR, RIBL, RIBR)	7.6%	${{\bf{D}}}_{{\bf{1}}}$	$< 1{0}^{-6}$
*Pseudosymmetry-Backward* *chemical* *synapse*			
(VA2, VA3, VA4, VA5)	4.5%	${{\bf{D}}}_{{\bf{1}}}$	0.002
(VA8, VA9)	0.8%	${{\bf{C}}}_{{\bf{2}}}$	$9\times 1{0}^{-5}$
(DA5, DA8, DA9, VA6, VA11)	10.8%	${{\bf{S}}}_{{\bf{5}}}$	$< 1{0}^{-6}$
(AVAL, AVAR)	21.5%	${{\bf{C}}}_{{\bf{2}}}$	$4\times 1{0}^{-6}$
(AVEL, AVER)	15.5%	${{\bf{C}}}_{{\bf{2}}}$	$8\times 1{0}^{-5}$
(AVDL, AVDR)	24.5%	${{\bf{C}}}_{{\bf{2}}}$	0.004
(DA1, DA2, DA3, DA4)	2.3%	${{\bf{S}}}_{{\bf{4}}}$	$4\times 1{0}^{-6}$
(VA10, DA6, DA7)	3.8%	${{\bf{S}}}_{{\bf{3}}}$	0.002

For each subgroup we show the uncertainty constant $\varepsilon$, which is below the 25% uncertainty given by the animal to animal experimental variability, and therefore the pseudosymmetries have biological significance. The provided *p*-value indicates that the pseudosymmetries have also statistical significance

### Symmetry group factorization into normal subgroups

The crucial property of the symmetry group ${{\bf{F}}}_{{\bf{gap}}}$ is its factorization into smaller ‘*normal subgroups*’. Its importance derives from the fact that these normal subgroups match the known broad functional categories of neurons involved in locomotion, such as command interneurons, motor and touch neurons^25^. A ‘*subgroup*’ ${\bf{H}}$ of a group ${\bf{G}}$ is a subset of transformations of ${\bf{G}}$ which forms itself a group, i.e., the transformations satisfy the four axioms of a group.

To understand what a normal subgroup is, we consider, for instance, the automorphism that exchanges the motor neurons *σ*:(VB2, DB3, DB2, VB1) $\leftrightarrow$ (DB1, VB4, VB5, VB6) and forms (with the identity) the dihedral group ${{\bf{D}}}_{{\bf{1}}}$ (Fig. 2a, b). Importantly, this automorphism acts independently only on neurons (VB2, DB3, DB2, VB1, DB1, VB4, VB5, VB6), and leaves the rest of the neurons of the connectome intact. Likewise, the automorphism $\tau{\!\!}:{\!\!}{\mathrm{VB7}} \leftrightarrow {\mathrm{VB3}}$ forms another group by itself, called the cyclic group of order 2, ${{\bf{C}}}_{{\bf{2}}}$, and also acts independently on this set of neurons and not on others.

The property of acting independently on a subset of neurons means that ${{\bf{D}}}_{{\bf{1}}}$ (and ${{\bf{C}}}_{{\bf{2}}}$) forms itself a smaller group, called a ‘*normal subgroup*’ inside the full symmetry group ${{\bf{F}}}_{{\bf{gap}}}$. More formally, a subgroup ${\bf{H}}$ is said to be normal in a group ${\bf{G}}$ if and only if ${\bf{H}}$ commutes with every element $g\in {\bf{G}}$, i.e., $[g,{\bf{H}}]=g{\bf{H}}-{\bf{H}}g=0$ The formal definition of subgroup and normal subgroup are explained in Supplementary Note 4, see ref. ^12^.

This property implies that the group ${{\bf{F}}}_{{\bf{gap}}}$ can be factorized in a unique way as a direct product of its two normal subgroups as: ${{\bf{D}}}_{{\bf{1}}}\times {{\bf{C}}}_{{\bf{2}}}$ (definition of factorization of a group in Supplementary Note 4, see ref. ^12^). The significance of the normal subgroup is that the normal transformations identify a unique and non-overlaping subset of neurons that are moved by each normal subgroup. This set of neurons are called the ‘*sector*’ associated with the normal subgroup. Since each normal subgroup acts only on an independent sector, the factorization of groups into normal subgroups leads also to a partition of neurons into unique disjoint sectors.

In simple terms, this means that when an automorphism in a normal subgroup is applied to the network, only the neurons in the sector of the normal subgroup are permuted, while the rest of the neurons that are outside the sector are not affected. Thus, we say that the normal subgroup automorphisms act only on the neurons belonging to its sector providing a unique separation and classification of the neurons and the associated factorization of the symmetry group. This factorization is mathematically analogous to the unique factorization of natural numbers into primes, and this notion is extended to group theory for those finite groups that can be factorized into ‘prime’ normal subgroups, as it is the case of the connectome.

The symmetry group ${{\bf{F}}}_{{\bf{gap}}}$ is factorized as a direct product of $6$ normal subgroups as:

4 $${{\bf{F}}}_{{\bf{gap}}}\ =\ [{{\bf{C}}}_{{\bf{2}}}\times {{\bf{C}}}_{{\bf{2}}}]\,\times \,[{{\bf{S}}}_{{\bf{5}}}\times {{\bf{D}}}_{{\bf{1}}}\times {{\bf{C}}}_{{\bf{2}}}\times {{\bf{C}}}_{{\bf{2}}}]\,.$$

Each subgroup acts on a non-overlapping independent sector of neurons as indicated in Fig. 2 (see also Supplementary Note 5). Table 1 lists the uncertainty constant for each subgroup of pseudosymmetries indicating that all $\varepsilon$ are small and below the experimental upper limit 25%^5,9,16,17^.

The factorization of the symmetry group ${{\bf{F}}}_{{\bf{gap}}}$ in Eq. (4) is significant because it determines a partition of the circuit into sectors that match specific categories of neurons^25^. To define the functional categories or classes of neurons we follow the literature where functions have been determined experimentally and compiled at the WormAtlas^25^. Broad functional categories of neurons are provided at http://www.wormatlas.org/hermaphrodite/nervous/Neuroframeset.html, Chapter 2.2. A classification for every neuron into four broad neuron categories follows: (1) motor neurons, (2) sensory neurons, (3) interneurons, and (4) polymodal neurons. A function is assigned to each neuron based on this experimental classification into neuron categories. This classification is displayed in Supplementary Tables 1 and 2, and discussed in Supplementary Note 6. These categories represent the ground truth to test the predictions of our theory.

Specifically, the factor $[{{\bf{C}}}_{{\bf{2}}}\times {{\bf{C}}}_{{\bf{2}}}]$ corresponds to the command interneuron category and comprises command interneurons which drive the forward locomotion: AVBL, ABVR, and RIBL, RIBR. The entire motor class is associated to an entire independent factor $[{{\bf{S}}}_{{\bf{5}}}\times {{\bf{D}}}_{{\bf{1}}}\times {{\bf{C}}}_{{\bf{2}}}\times {{\bf{C}}}_{{\bf{2}}}]$, and includes all motor neurons innervating the muscle cells responsible for the undulatory motion of *C. elegans*.

**Fig. 3:**
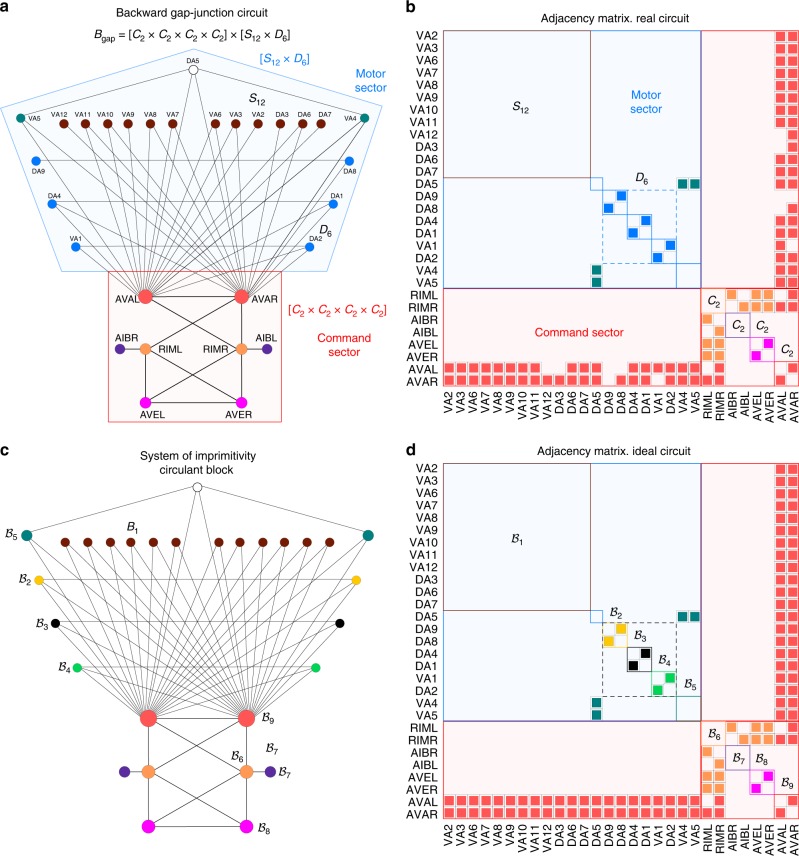
Symmetry group ${{\bf{B}}}_{{\bf{gap}}}$ of the backward gap-junction circuit. **a** The real circuits and (**b**) its adjacency matrix. The symmetry group is factorized as a direct product of normal subgroups: ${{\bf{B}}}_{{\bf{gap}}}=\ [{{\bf{C}}}_{{\bf{2}}}\times {{\bf{C}}}_{{\bf{2}}}\times {{\bf{D}}}_{{\bf{1}}}]\,\times [{{\bf{S}}}_{{\bf{12}}}\times {{\bf{D}}}_{{\bf{6}}}\times {{\bf{C}}}_{{\bf{2}}}]$, which leads to a partition of neurons in two sectors that match the command and motor sectors known experimentally, as indicated. **c** Ideal circuit and (**d**) adjacency matrix highlighting the primitive and imprimitive blocks and their circulant structures from ${{\mathcal{B}}}_{1}$ to ${{\mathcal{B}}}_{9}$

**Fig. 4:**
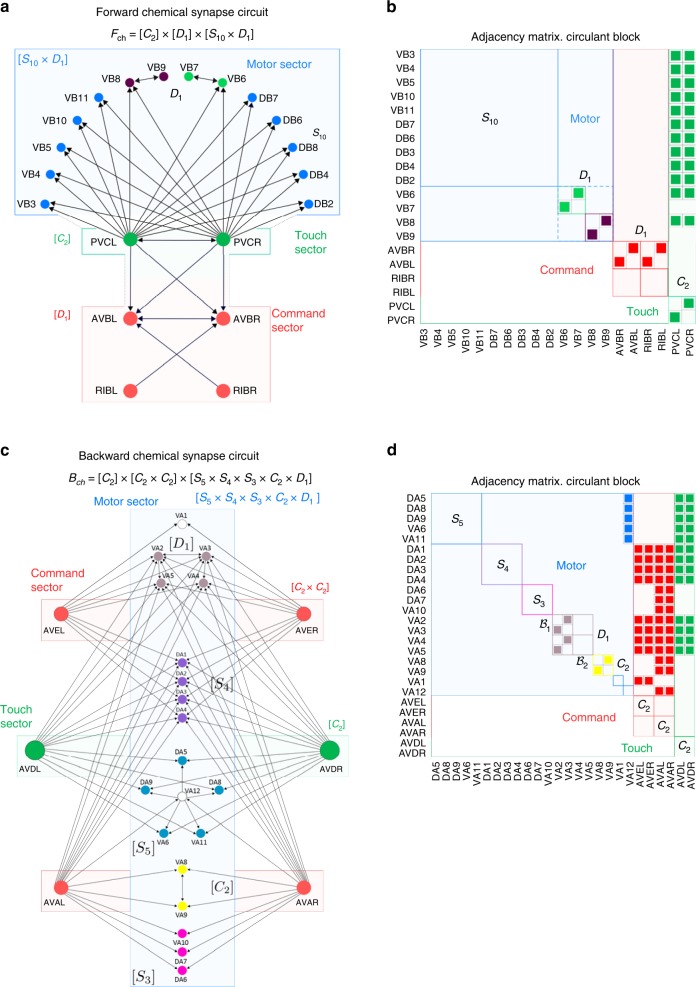
Symmetry groups ${{\bf{F}}}_{{\bf{ch}}}$ and ${{\bf{B}}}_{{\bf{ch}}}$ of the chemical synapse forward and backward circuits. **a** Forward locomotor chemical synapse circuit and (**b**) its adjacency matrix (ideal circuits, real circuits in Supplementary Figs. 5 and 6). The symmetry group ${{\bf{F}}}_{{\bf{ch}}}$ is factorized into the direct product of command, motor, and touch subgroups as ${{\bf{F}}}_{{\bf{ch}}}=\,{{\bf{C}}}_{{\bf{2}}}\,\times \,[{{\bf{D}}}_{{\bf{1}}}]\,\times \,[{{\bf{S}}}_{{\bf{10}}}\times {{\bf{D}}}_{{\bf{1}}}]$, which, in turn, split up the circuits into independent sectors of neurons matching different functions and include also the neuron touch class PVC (forward) and AVD (backward). **c** The backward circuit factorizes as ${{\bf{B}}}_{{\bf{ch}}}\,=\,{{\bf{C}}}_{{\bf{2}}}\,\times \,[{{\bf{C}}}_{{\bf{2}}}\times {{\bf{C}}}_{{\bf{2}}}]\,\times \,[{{\bf{S}}}_{{\bf{5}}}\times {{\bf{S}}}_{{\bf{4}}}\times {{\bf{S}}}_{{\bf{3}}}\times {{\bf{D}}}_{{\bf{1}}}\times {{\bf{C}}}_{{\bf{2}}}\times {{\bf{C}}}_{{\bf{2}}}].$ We show the ideal circuit and (**d**) its adjacency matrix. For simplicity we plot only the interneurons that connect to the motor neurons. Full circuit in SM Fig. 6. All neurotransmitters are cholinergic and excitatory (ACh) except for RIM which uses neurotransmitter Glutamate and Tyramine and AIB which is glutamatergic (see Supplementary Note 6). These different types of synaptic interactions respect the symmetries of the circuits, see Supplementary Note 5

Applying the same symmetry procedure, we find that all forward/backward gap-junction/chemical synapse circuits form symmetry groups, and these groups can be factorized into normal subgroups in the same way as in Eq. (4) (see Figs. 3 and 4 and Supplementary Note 5 for details). The correspondence between neuron sectors from group theory and known categories of neurons occurs consistently across all circuits, and further includes a subgroup related to touch sensitivity^6^ in the forward (PVCL, PVCR, Fig. 4a) and backward (AVDL, AVDR, Fig. 4c) chemical synaptic circuits. The full list of sectors, normal subgroups and uncertainty constants of the pseudosymmetries are provided in Table 1.

The normal subgroups partition the connectome into non-overlapping functional sector of neurons, thus realizing the segregation of function. At the same time, the sectors remain connected in the connectome without breaking the symmetries, thus fulfilling the integration of function into a globally connected network. Thus, the symmetric subgroup organization of the connectome provides an elegant solution to the conundrum of functional specialization in the presence of a global integration of information necessary for efficient coherent function^26^, a profound issue in neuroscience

While group factorization can distinguish different classes of neurons, this distinction may also be seen in some cases by directly looking at the adjacency matrix: for instance in Fig. 2b the AVB interneurons are heavily connected hub neurons which could be, in principle, also distinguished by any connectivity measure. That is, the neurons AVBL and AVBR are hubs with large degree $k=18$ and are easily distinguished from the rest of the neurons which have generally smaller degree. However, in general, having the same degree does not imply that the neurons belong to the same subgroup. Thus, the connectivity measure alone may not fully capture the symmetry groups that we find.

For instance, neurons can be in the same sector subgroup and at the same time could, in principle, have different degree. This situation is seen for example in the neurons of the forward motor sector subgroup ${{\bf{D}}}_{{\bf{1}}}$ in Fig. 2a, c. In the circuit of Fig. 2c, the neurons in ${{\bf{D}}}_{{\bf{1}}}$ have different degree: VB5, DB2 VB4 and DB3 with $k=5$, VB1 and VB6 with $k=3$, VB2 and DB1 with $k=8$. Thus, even though these neurons have different degree, they belong to the same subgroup and functional class: the motor sector subgroup ${{\bf{D}}}_{{\bf{1}}}$. In general, the degree alone is not enough to separate the neurons in subgroups and known classes.

Furthermore, Fig. 2b shows that the pair (VB8, VB9) has the same connectivity as the pair (RIBL, RIBR), and thus they could be classified in the same category as either motor neuron (with VB) or interneurons (with RIB). If we consider the neurons unweighted they merge into the same subgroup and they should perform the same function. However, considering the weights, there is an asymmetry, since both, VB9 and VB8 have 6 and 7 connections to AVBL and AVBR respectively, while RIBL and RIBR have one connection each to both neurons (see Supplementary Fig. 3). Indeed, the WormAtlas classifies RIBL and RIBR as interneurons^25^, thus, we classify these pairs of neurons in different classes. The asymmetry in the weights might the reason why the experiments compiled at the WormAtlas find that these two set of neurons may work in different categories: motor and interneuron. In general, it is possible for a neuron to be involved in multiple functions. The case of polymodal neurons can be treated theoretically by generalizing the direct-product factorization to semidirect-product factorization of normal and non-normal subgroups. Semi-direct product factorization could capture overlapping sectors of neurons and multi-functionality which are more prevalent across the connectome beyond locomotion.

### Statistical significance of the symmetry subgroups

To establish the degree to which the symmetries of the locomotion sub-circuits are statistically significant, we compare the symmetry subgroups against control random sub-circuits. Indeed, a high enough value of $\epsilon$ would yield an approximate symmetric version of any arbitrary circuit: a fully random non-symmetric connectome implies $\epsilon =1$, and a perfect symmetric one $\epsilon =0$. In between, all networks can be classified by their $\epsilon$-value. Thus, it is important that not only $\epsilon$ be smaller than the experimental variability $\epsilon {\,} < {\,}25 \%$, but also be statistically significant. Statistical metrics to evaluate the symmetries are *p*-value statistical tests to compare results with a randomized null model preserving the degree sequence. Specifically, the *p*-value of a pseudo-symmetry subgroup ${G}_{\epsilon }$ is defined as the probability to find a subgroup ${G}_{{\epsilon }^{* }}$ with ${\epsilon }^{* }\le \epsilon$ in a randomized circuit with the same degree sequence as the real circuit. The results of the p-values are summarized in Table 1 for each subgroup, showing that pseudosymmetry subgroups are, indeed, statistically significant.

### Comparison with other methods to find functional modules

It is interesting to compare the functional partition obtained by the symmetries of the connectome with typical modularity detection algorithms which are widely used to identify functional modules in biological networks^27^. Indeed, there is a large body of work which examines the connectivity of biological networks to algorithmically classify the constituent neurons into modules and compare those modules to known classifications. Therefore, below we investigate how symmetry detected sectors compare to existing algorithms such as modularity and community detection, and other centrality measures.

**Fig. 5:**
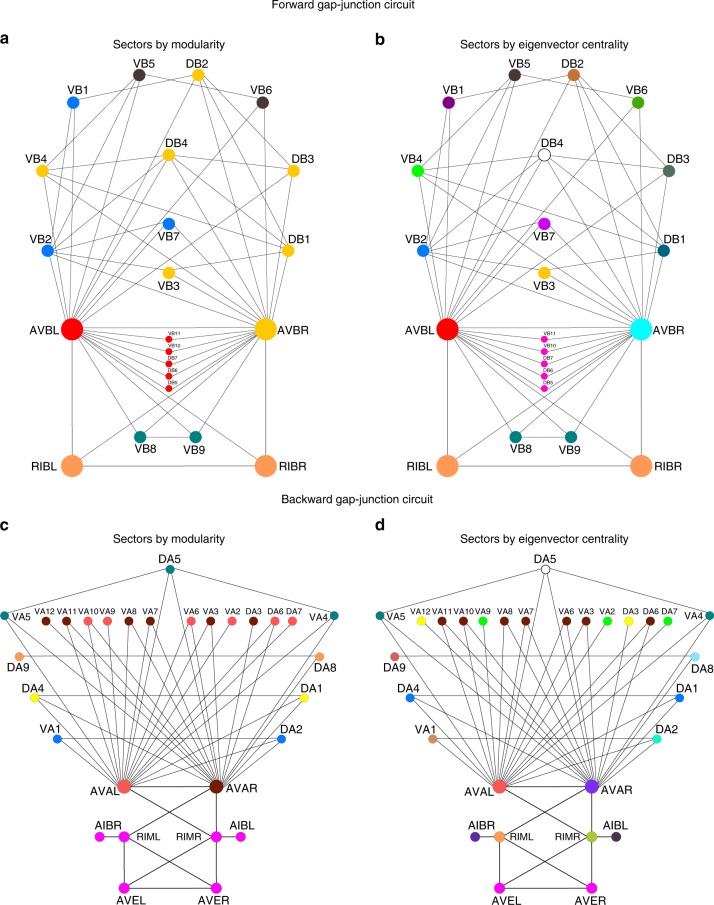
Symmetry vs. other methods. We compare the functional classes obtained from symmetries with modularity detection algorithms^27,28^ and a typical eigenvector centrality measure. **a** Forward gap-junction circuit classes obtained using modularity or community detection detection algorithm from ref. ^28^ and (**b**) using eigenvector centrality. **c** Backward gap-junction circuit modularity and (**d**) eigenvector centrality. Both measures, modular detection and centrality, do not capture the symmetries and functional classification of this connectome

We run the Louvain community detection algorithm^28^ on the forward and backward circuit and find the modular partition seen in Fig. 5. We find that modules identified by the Louvain algorithm do not generally capture the functional modules identified by symmetry subgroups, nor the experimental classification into neural functions.

Typically, the modularity algorithm assigns to the same functional module a hub-like interneuron AVBR together with its connected neurons in the motor sector (see Fig. 5a), since these neurons are all highly connected. Thus, the modularity algorithm will typically mix the interneuron and motor sectors. Symmetry factorization into normal subgroups, on the other hand, correctly classifies AVBL and AVBR separately from the VB and DB neurons in the motor sector, even though these sectors are well connected. Similar results are obtained when we use other network centralities: Fig. 5b, d show the modules obtained by ranking neurons according to eigenvector centrality. We find that such centrality measure does not capture the partition into symmetry sectors nor the functional classes.

### A recurrent and feedforward neural network made of blocks of imprimitivity and circulant matrices

The data analyzed so far indicate that there is still a more refined structure inside the broad functional categories of motor, command and touch, that requires further exploration. For instance, the motor class of forward gap junctions (Fig. 2) consists of 4 different normal subgroups: $[{{\bf{S}}}_{{\bf{5}}}\times {{\bf{D}}}_{{\bf{1}}}\times {{\bf{C}}}_{{\bf{2}}}\times {{\bf{C}}}_{{\bf{2}}}]$. Next, we show that the functionality of this finer structure can be systematically obtained through a more refined group theoretical concept of ‘*block of imprimitivity*’^12^, which identifies the fundamental processing units of the connectome and naturally leads to a novel functionality in terms of mechanism of neural coding.

A block of imprimitivity is a set of neurons that, under the action of the automorphisms of a subgroup, is completely mapped onto itself or it is mapped onto a completely disjoint set of neurons (formal definition of block of imprimitivity in Supplementary Note 7, see^12^, and Fig. 1f). For instance, consider the subgroup ${{\bf{D}}}_{1}$ of the forward gap-junction circuit (Fig. 2) which consists of the automorphism $\sigma \in {{\bf{D}}}_{{\bf{1}}}$ which acts on the sector (VB2, DB3, DB2, VB1, DB1, VB4, VB5, VB6). The subset of neurons highlighted in green in Fig. 2c, ${{\mathcal{B}}}_{1}=$ (VB2, DB3, DB2, VB1), forms a block of imprimitivity since $\sigma$ moves this set into a different one, highlighted in black, ${{\mathcal{B}}}_{2}=$ (DB1, VB4, VB5, VB6), which is the other block of imprimitivity of the sector and a conjugate block of ${{\mathcal{B}}}_{1}$. These two blocks form the so-called *system of imprimitivity*, a fundamental concept in group theory^12,29^. The other normal subgroups of the forward circuit do not have a nontrivial block system of imprimitivity, hence they are said to be *primitive* (Supplementary Note 7^12^).

The resulting block partition of each adjacency matrix is shown in Figs. 2d, 3d and 4b, d. These systems of imprimitivity identify new functionalities in each locomotion circuit. Specifically, we find that the system of imprimitivity of each locomotion circuit is formed by blocks represented by *circulant* matrices^13^. A circulant matrix is a square matrix where each row is a cycle shift to the right of the row above it, and wrapped around^13^ (see Methods Section for definition). In alignment with pseudosymmetries, the circulant matrices are interpreted as pseudocirculant matrices of the real circuit. A pseudocirculant matrix differs from a circulant matrix by a fraction $\varepsilon$ of their links. We note that this partition into blocks of imprimitivity is not unique. For instance, another possible block system corresponds to a partition made by the orbits.

Circulant matrices are well-known in the field of digital signal processing, recurrent and feedforward neural networks^4^ and cryptography, and are widely used as efficient linear filters to solve a variety of tasks in digital image processing, most notably as edge-detection and signal compression^4,30^, but also in tracking^31^, voice recognition, and computer vision^32^. Circulant matrices are the kernels of discrete convolutions and are used in discrete Fourier transform to solve efficiently systems of linear equations in nearly linear time^13^ that significantly speed up the $O({N}^{3})$ arithmetic complexity of Gaussian elimination.

We find different types of circulant matrices in the connectome which are, in turn, nested into larger block-circulant matrices (see definitions in Figs. 1b and 2d and Methods Section). Two circulant matrices occur consistently in all locomotion circuits and act as a ‘high-pass’ filter:

5 $${\mathcal{H}}={\rm{circ}}(0,1)=\left[\begin{array}{ll}0&1\\ 1&0\end{array}\right],$$

and a ‘low-pass’ filter:

6 $${\mathcal{L}}={\rm{circ}}(1,1)=\left[\begin{array}{ll}1&1\\ 1&1\end{array}\right].$$

The third type of circulant matrix represents a 4-cycle permutation:

7 $${\mathcal{F}}={\rm{circ}}(0,1,0,1)=\left[\begin{array}{rcll}0&1&0&1\\ 1&0&1&0\\ 0&1&0&1\\ 1&0&1&0\end{array}\right],$$

and acts on the blocks of imprimitivity ${{\mathcal{B}}}_{1}$ and ${{\mathcal{B}}}_{2}$ in the motor sector of the forward gap-junction circuit (Figs. 2c, d). Intuitively, each circulant matrix represents a cycle embedded in the subgroup sector as seen in Fig. 2c for ${{\mathcal{B}}}_{1}{\!\!}:$VB2 $\to$ DB3 $\to$ DB2 $\to$ VB1 $\to$ VB2. In the same figure we see the 4-cycle of the conjugate block ${{\mathcal{B}}}_{2}$.

The $2\times 2$ circulant matrix ${\mathcal{H}}$ in Eq. 5 is quite ubiquitous and corresponds to a 2-cycle (or transposition). For instance, the 2-cycle VB8 $\to$ VB9 $\to$ VB8 in the forward gap junction circuit Fig. 2c forms a circulant matrix of the form given by ${\mathcal{H}}$. This is also a block of imprimitivity, since this block is the only one inside the subgroup ${{\bf{C}}}_{{\bf{2}}}$. Subgroup ${{\bf{S}}}_{{\bf{5}}}$ also forms a circulant matrix, although a trivial one in this case since all its elements are zero.

It is interesting to see that the circulant matrices are nested into an structure of block-circulant matrices (see Methods Section for definition), suggesting a hierarchical organization of building blocks in the connectome. Typical block-circulant matrices are of the form^13^:

8 $${\mathcal{BC}}\ =\ {\rm{bcirc}}({\mathcal{H}},{\mathcal{L}})\ =\ \left[\begin{array}{ll}{\mathcal{H}}&{\mathcal{L}}\\ {\mathcal{L}}&{\mathcal{H}}\end{array}\right]\ =\ \left[\begin{array}{rcll}0&1&1&1\\ 1&0&1&1\\ 1&1&0&1\\ 1&1&1&0\end{array}\right].$$

For instance, this block-circulant matrix appears in the command sector of the forward gap junction circuit between the neurons RIBL, RIBR, AVBL, AVBR. This is seen in Fig. 1b and also in Fig. 2d. It is interesting to note that when we analyze the group structure of the interneuron only circuit of gap junctions, then we find the group structure shown in Fig. 1b. When we integrate this circuit in the full forward circuit, then this group becomes a system of imprimitivity shown as ${{\mathcal{B}}}_{6}$ and ${{\mathcal{B}}}_{7}$ in Fig. 2d. This is a block-circulant matrix made itself by circulant matrices forming a nested hierarchical structure. This hierarchical nestedness is repeated across all the connectome.

A block-circulant structure is formed by the imprimitive blocks ${{\mathcal{B}}}_{1}$ and ${{\mathcal{B}}}_{2}$ in the same forward gap junction circuit, Fig. 2d. In this case, we have:

9 $${A}_{1}={\mathcal{F}}={\rm{circ}}(0,1,0,1) 	=\left[\begin{array}{rcll}0&1&0&1\\ 1&0&1&0\\ 0&1&0&1\\ 1&0&1&0\end{array}\right],\ \ {\rm{and}}\ \\ {A}_{2} 	=\left[\begin{array}{rcll}0&0&0&1\\ 0&0&0&0\\ 0&1&0&0\\ 0&0&0&0\end{array}\right],$$

and both ${{\mathcal{B}}}_{1}$ and ${{\mathcal{B}}}_{2}$ combine into a block-circulant matrix of the form:

10 $${\mathcal{B}}C={\rm{bcirc}}({A}_{1},{A}_{2}).$$

Also, ${{\mathcal{B}}}_{6}$ and ${{\mathcal{B}}}_{7}$ in the backward gap junction circuit of Fig. 3d composed of neurons AIBL, AIBR, RIML, RIMR form a block-circulant matrix

11 $${\mathcal{B}}C={\rm{bcirc}}({A}_{1},{A}_{2})$$

with

12 $${A}_{1}={\rm{circ}}(0,0)\,\ \ {\rm{and}}\ \ {A}_{2}\ =\ {\rm{circ}}(1,0)\ =\ \left[\begin{array}{ll}1&0\\ 0&1\end{array}\right].$$

These results suggest that we can think of the connectome as a feedforward network made of interneurons that feeds a recurrent network in the motor system^4^ made of a system of sensing operators, each represented by an imprimitive block with a circulant structure. Such a feed-forward and recurrent network architecture is universally seen across many neural systems and it is used as a model of the receptive fields in the primary visual cortex^4^. Such a system can be modeled by a feedforward matrix ${\bf{W}}$ and a recurrent network ${\bf{M}}$ processing the input activity ${\bf{u}}$ to the output ${\bf{v}}$ as a linear filter, see Dayan and Abbott^4^:

13 $$\tau \frac{d{\bf{v}}}{dt}=-{\bf{v}}+{\bf{M}}{\bf{v}}+{\bf{W}}{\bf{u}},$$

where $\tau$ is a time characteristic. The crucial property of this system is that the matrix ${\bf{M}}$ contains loops in the network.

For instance, in the case of the gap junction forward circuit (Fig. 2), the AVBL and AVBR interneurons act as the input layer ${\bf{u}}={({u}_{{\rm{ABV}}{\rm{L}}},{u}_{{\rm{ABV}}{\rm{R}}})}^{T}$ which is first processed by the feedforward matrix represented by a fully connected matrix:

14 $${\bf{W}}\ =\ \left[\begin{array}{ll}1&1\\ 1&1\\ 1&1\\ 1&1\end{array}\right],$$

whose output is then processed by the recurrent network in the motor sector by, for instance, processing the signal in the motor neurons of the imprimitivity block ${{\mathcal{B}}}_{1},{\bf{v}}={({v}_{{\rm{V}}\; \rm{{B}}2},{v}_{{\rm{DB}}3},{v}_{{\rm{DB}}2},{v}_{{\rm{V}}\; \rm{{B}}1})}^{T}$ by the recurrent circulant matrix ${\bf{M}}={\mathcal{F}}={\rm{circ}}(0,1,0,1)$ from Eq. (8). The same signal processing occurs in the feedforward and recurrent network formed by the conjugate motor imprimitive block ${\mathcal{B}}_{2}$. Similar structure is seeing in the backward circuit Fig. 3 with AVAL-AVAR feed-forwarding information into the recurrent circulant blocks in the motor sector. The chemical circuits also contain such a feed-forward and recurrent structure: PVCL-PVCR feeds the forward motor circulant blocks (Fig. 4a) and AVE-AVD-AVA feed the backward motor circulant blocks (Fig. 4c).

Using the language of signal processing in computational neuroscience, these recurrent networks are analogous to the core of receptive fields that process information in the visual cortex, see Dayan & Abbott^4^. For instance a widely used filter in signal processing is the edge-detector^4,30^ which employs a circulant matrix defined by ${\bf{M}}={\rm{circ}}(0,1,-1,0,\cdots \ ,0)$ to compute a ‘derivative’ of the spatial signal and detect sharp edges^4^. Another typical computation is performed by a circulant matrix ${\bf{M}}={\rm{circ}}(0,1,-2,1,0,\cdots \ ,0)$ to represent a second derivative of the signal, and so on.

In the case of the connectome, one possible interpretation of the purpose of the found circulant filters is to separate one band of frequencies from another and perform signal compression. The high-pass filter ${\mathcal{H}}$ is used to block the low frequency content of the neural signal, while the low-pass filter eliminates the high frequencies. The ${\mathcal{F}}$ matrix is a translational invariant filter to sample the signal as a way of reducing the size of the signal (compression) without overly reducing its information content to process the undulatory motion of locomotion according to its eigenvalues.

Roughly speaking, the filter ${\mathcal{H}}$ measures the self-similarity on either side of the center point and the output will be maximal when each the two points are equal to each other. The filter ${\mathcal{F}}$ operates on the inputs of the imprimitive systems of the forward circuit. The fact that this matrix appears only in the forward circuit suggests that it might be an important controller in the undulatory motion. This can be seen from the eigenvalues ${\lambda }_{i}$ of this circulant matrix and their eigenvectors ${{\bf{v}}}_{{\bf{i}}}$:

15 $${\lambda }_{1}	=-2,\quad\;{{\bf{v}}}_{{\bf{1}}}=\frac{1}{2}(-1,1,-1,1),\\ {\lambda }_{2}	=2, \quad\quad{{\bf{v}}}_{{\bf{2}}}=\frac{1}{2}(1,1,1,1),\\ {\lambda }_{3}	=0,\quad\quad{{\bf{v}}}_{{\bf{3}}}=\frac{1}{\sqrt{2}}(0,-1,0,1),\\ {\lambda }_{4}	=0,\quad\quad{{\bf{v}}}_{{\bf{4}}}=\frac{1}{\sqrt{2}}(-1,0,1,0),$$

which determine the solution of Eq. 13^4^ and act by filtering out two modes and allow oscillations between ${\lambda }_{1}$ and ${\lambda }_{2}$. Thus, the circulant blocks act as information processing units in the recurrent network that are basically filters to perform specific signal processing operations (see Supplementary Note 8).

The association of the circulant processing units with the blocks of imprimitivity completes the operational definition of the locomotor function determined by the decomposition properties of its symmetry group, and in turn, unveil and classify hitherto hidden mechanisms of the neural code. The existence of the predicted blocks can be directly tested in future experiments by measuring how the imprimitive blocks process the neural signal in real time according to their circulant filters.

## Discussion

Overall, the structure-function relation in the connectome can be seen as a refining process of nested symmetry building blocks. The primary building blocks are defined through the mechanism of direct product factorization of normal subgroups and provide a rigorous characterization of the network connectivity structure, and a simple interpretation of its major functions into neural classes. These major sectors are comprised of secondary topological structures involved in signal processing which refine the primary normal subgroups into irreducible blocks of imprimitivity.

The factorization of the symmetry groups of the connectome has its analogy with integers and primes as every integer can be factorized into a unique product of prime numbers as stated in the fundamental theorem of arithmetic. This factorization is also analogous to that of the Standard Model of particle physics^29^. In theoretical physics, automorphisms describe the symmetries of elementary particles and forces^29,33^, as well as atoms, molecules and phases of matter^34^. For example, fundamental forces in particle physics are based on symmetry principles incorporated through a description of the gauge symmetry group of the Lagrangian factorized into three subgroups as ${\rm{U}}(1)\times {\rm{SU}}(2)\times {\rm{SU}}(3)$, where ${\rm{SU}}(N)$ is the special unitary group of $N\times N$ unitary matrices with determinant 1, and ${\rm{U}}(1)$ is the group consisting of all complex numbers with absolute value 1. In this case, each subgroup determines a different force, namely the electroweak and strong forces, and the generators of these symmetry subgroups are the particles. Analogously, the functions of locomotion are based on the symmetries of the connectome through the symmetry group which is factorized in general as ${\rm{T}}\times {\rm{C}}\times {\rm{M}}$ where each symmetry subgroup determines a different function. For instance, the symmetry group of the chemical forward circuit splits as: ${{\bf{F}}}_{{\bf{ch}}}={{\mathbb{T}}}_{{{\bf{F}}}_{{\bf{ch}}}}\times {{\mathbb{C}}}_{{{\bf{F}}}_{{\bf{ch}}}}\times {{\mathbb{M}}}_{{{\bf{F}}}_{{\bf{ch}}}}$.

In a milestone in the history of mathematics, all finite simple groups have been discovered and classified into 3 major classes: cyclic, alternating or Lie type plus 26 extra classes of rare sporadic groups^35^. Out this variety, the locomotion connectome contains only cyclic groups. It would be fascinating to discover other naturally occurring simple groups for other functions in different biological networks. Results presented elsewhere indicate that symmetries extend to the full connectome and also to genetic networks^36^, and they are naturally related to neural synchronization. Thus, the principle of symmetry provides a rigorous mathematical characterization of the structural and functional organization of connectomes down to their information-processing units. This hierarchical symmetric architecture may also serve as guidance to design more efficient artificial neural networks inspired by natural systems.

## Methods

### Circulant and block-circulant matrices in digital signal processing and the connectome

We find that the system of imprimitivity of the locomotion circuits is comprised of a specific type of blocks, which are represented by circulant matrices^13^, https://en.wikipedia.org/wiki/Circulant_matrix.

It is worth noting that there is a priori no reason for the occurrence of this specific type of matrices in the system of imprimitivity. That is, a symmetry group may have a system of imprimitivity that is not composed of circulant matrices. Thus, there are two independent results: first, the connectome is broken down into a system of imprimitivity. Second, the imprimitive blocks have the shape of circulant matrices and block circulant matrices.

A circulant matrix ${P}_{\ell }$ of order $\ell$ is a square matrix of the form^13^:

16 $${P}_{\ell }\,=\,{\rm{circ}}({c}_{1},{c}_{2},\ldots ,{c}_{\ell })\ =\ \left[\begin{array}{lllll}{c}_{1}&{c}_{2}&{c}_{3}&\ldots &{c}_{\ell }\\ {c}_{\ell }&{c}_{1}&{c}_{2}&\ldots &{c}_{\ell -1}\\ \cdot &\cdot &&&\cdot \\ \cdot &\cdot &&&\cdot \\ {c}_{2}&{c}_{3}&{c}_{4}&\ldots &{c}_{1}\end{array}\right].$$

The elements of each row are the same as those from the previous row, but are shifted one position to the right and wrapped around. The circulant matrix is thus determined by the first row or column and therefore it is denoted by^13^: ${P}_{\ell }\,=\,{\rm{circ}}({c}_{1},{c}_{2},\ldots ,{c}_{\ell })$.

We also find block-circulant matrices in the connectome which are defined as follows. Block-circulant matrices are an extension of circulant matrices where the elements ${c}_{i}$ are now replaced by matrices themself ${A}_{i}$. Let ${A}_{1},{A}_{2},\ldots ,{A}_{m}$ be square matrices of order $n$. A block-circulant matrix of order $mn$ is the form^13^:

17 $${\mathcal{BC}}\,=\,{\rm{bcirc}}({A}_{1},{A}_{2},\ldots ,{A}_{m})\ =\ \left[\begin{array}{lllll}{A}_{1}&{A}_{2}&{A}_{3}&\ldots &{A}_{m}\\ {A}_{m}&{A}_{1}&{A}_{2}&\ldots &{A}_{m-1}\\ \cdot &\cdot &&\cdot \\ \cdot &\cdot &&\cdot \\ {A}_{2}&{A}_{3}&{A}_{4}&\ldots &{A}_{1}\end{array}\right],$$

and when $n=1$, the block-circulant becomes a circulant matrix. The matrices ${A}_{i}$ may not need to be necessarily circulant. However, the connectome presents only circulant matrices as ${A}_{i}$, thus creating a hierarchical nested structure of circulant blocks made of circulant matrices themself.

The graph that results from a circulant matrix is called a circulant graph, https://en.wikipedia.org/wiki/Circulant_graph. Circulant matrices are determined by the first row and every row is the cyclic shift of the row above it. A circulant matrix is a special kind of Toeplitz matrix with the additional property that ${c}_{i}={c}_{i+\ell }$^13^.

Repeated application of ${P}_{\ell }$ on itself generates an abelian group called cyclic group of order $\ell$, denoted as ${{\bf{C}}}_{{\boldsymbol{\ell }}}$. Moreover, any subgroup of ${{\bf{C}}}_{{\boldsymbol{\ell }}}$ is also cyclic. The important point is that whenever the symmetry group of a network contains a circulant permutation matrix like ${P}_{\ell }$ in Eq. (16), then the adjacency matrix $A$, or a piece of it, inherits from ${P}_{\ell }$ the same circulant structure.

In the locomotion neural circuits studied in this work, we find 3 types of circulant matrices: ${\mathcal{H}},{\mathcal{L}},$ and ${\mathcal{F}}$. In the language of signal processing, the matrix ${\mathcal{H}}$ is a spatial high-pass filter, used to block the low frequency content of the signal; and ${\mathcal{L}}$ is a spatial low-pass filter, which instead eliminates the high frequencies. These are the two most common linear filters used in image processing. The filter ${\mathcal{F}}$ is the kernel of the fast Fourier transform (see Supplementary Note 8). It can be thought as a translational invariant filter to sample the signal as a way of reducing the size of the signal without overly reducing its information content. While ${\mathcal{H}}$ and ${\mathcal{L}}$ appear consistently across all circuits, the circulant ${\mathcal{F}}$ matrix occurs only in the forward gap-junction circuit. The low pass filter selects the ‘bulk’ of the information, while the ‘high-pass’ picks out finer details.

This structure shows how the connectome acts as a signal processing network within a hierarchical structure that starts at the symmetry group level, which is then broken down into subgroups and further broken into the system of imprimitivity which represents the irreducible building blocks.

### Reporting summary

Further information on research design is available in the Nature Research Reporting Summary linked to this article.

## Supplementary information

Reporting Summary

Supplementary Information

## Data Availability

Connectome data are available in the public domain at http://www.wormatlas.org and codes at http://www.kcorelab.org and http://github.com/Makselab.
