# Supplementary material for: Symmetry group factorization reveals the structure-function relation in the neural connectome of Caenorhabditis elegans
Source: Nat Commun. 2019 Oct 31;10:4961. doi: 10.1038/s41467-019-12675-8 (PMC6823386; doi:10.1038/s41467-019-12675-8)
Supplement: Supplementary file 1 — Reporting Summary [file 41467_2019_12675_MOESM1_ESM.pdf]

nature research

Corresponding author(s): Hernan A. Makse

Last updated by author(s): Jul 16, 2109

Reporting Summary

Nature Research wishes to improve the reproducibility of the work that we publish. This form provides structure for consistency and transparency in reporting. For further information on Nature Research policies, see Authors & Referees and the Editorial Policy Checklist.

Statistics

For all statistical analyses, confirm that the following items are present in the figure legend, table legend, main text, or Methods section.

n/a	Confirmed	
☐	☒	The exact sample size (*n*) for each experimental group/condition, given as a discrete number and unit of measurement
☒	☐	A statement on whether measurements were taken from distinct samples or whether the same sample was measured repeatedly
☐	☒	The statistical test(s) used AND whether they are one- or two-sided<br>*Only common tests should be described solely by name; describe more complex techniques in the Methods section.*
☒	☐	A description of all covariates tested
☒	☐	A description of any assumptions or corrections, such as tests of normality and adjustment for multiple comparisons
☐	☒	A full description of the statistical parameters including central tendency (e.g. means) or other basic estimates (e.g. regression coefficient) AND variation (e.g. standard deviation) or associated estimates of uncertainty (e.g. confidence intervals)
☐	☒	For null hypothesis testing, the test statistic (e.g. *F*, *t*, *r*) with confidence intervals, effect sizes, degrees of freedom and *P* value noted<br>*Give P values as exact values whenever suitable.*
☒	☐	For Bayesian analysis, information on the choice of priors and Markov chain Monte Carlo settings
☒	☐	For hierarchical and complex designs, identification of the appropriate level for tests and full reporting of outcomes
☐	☒	Estimates of effect sizes (e.g. Cohen's *d*, Pearson's *r*), indicating how they were calculated

Our web collection on statistics for biologists contains articles on many of the points above.

Software and code

Policy information about availability of computer code

Data collection	Data used in this study is available in in the public domain at http://www.wormatlas.org
Data analysis	In this study we used the software Gephi - The Open Graph Viz Platform - v 0.9.2, and proprietary code written in C++ language available available at http://www.kcorelab.org

For manuscripts utilizing custom algorithms or software that are central to the research but not yet described in published literature, software must be made available to editors/reviewers. We strongly encourage code deposition in a community repository (e.g. GitHub). See the Nature Research guidelines for submitting code & software for further information.

Data

Policy information about availability of data

All manuscripts must include a data availability statement. This statement should provide the following information, where applicable:

- Accession codes, unique identifiers, or web links for publicly available datasets
- A list of figures that have associated raw data
- A description of any restrictions on data availability

Data is available in the public domain at http://www.wormatlas.org, and codes are available at http://www.kcorelab.org.

Field-specific reporting

Please select the one below that is the best fit for your research. If you are not sure, read the appropriate sections before making your selection.

☒ Life sciences ☐ Behavioural & social sciences ☐ Ecological, evolutionary & environmental sciences

Life sciences study design

All studies must disclose on these points even when the disclosure is negative.

Sample size	The sample analyzed in this study is the most updated connectome of the C.elegans comprising 302 neurons, which is available in public domain at http://www.wormatlas.org
Data exclusions	No data were excluded from the analysis.
Replication	We analyzed data collected in previous experiments, which established the reproducibility of the C.elegans neural wiring diagram within a 25% variation across animals.
Randomization	To establish the degree to which the symmetries of the locomotion sub-circuits are statistically significant, we compared the symmetry subgroups against control random sub-circuits. Statistical metrics to evaluate the symmetries are p-value statistical tests to compare results with a randomized null model preserving the degree sequence.
Blinding	Blinding was not relevant to our study, since we analyzed data collected in previous experiments and established in the scientific literature.

Reporting for specific materials, systems and methods

We require information from authors about some types of materials, experimental systems and methods used in many studies. Here, indicate whether each material, system or method listed is relevant to your study. If you are not sure if a list item applies to your research, read the appropriate section before selecting a response.

Materials & experimental systems

n/a	Involved in the study
☒	☐ Antibodies
☒	☐ Eukaryotic cell lines
☒	☐ Palaeontology
☒	☐ Animals and other organisms
☒	☐ Human research participants
☒	☐ Clinical data

Methods

n/a	Involved in the study
☒	☐ ChIP-seq
☒	☐ Flow cytometry
☒	☐ MRI-based neuroimaging
