# Supplementary material for: Symmetry group factorization reveals the structure-function relation in the neural connectome of Caenorhabditis elegans
Source: Nat Commun. 2019 Oct 31;10:4961. doi: 10.1038/s41467-019-12675-8 (PMC6823386; doi:10.1038/s41467-019-12675-8)
Supplement: Supplementary file 2 — Supplementary Information [file 41467_2019_12675_MOESM2_ESM.pdf]

Supplementary Information

Symmetry group factorization reveals the structure-function relation in the neural connectome of *Caenorhabditis elegans*

Flaviano Morone and Hernán A. Makse

Supplementary Note 1 - *C. elegans* connectome

We downloaded the most updated connectome of the hermaphrodite worm *Caenorhabditis elegans* (*C. elegans*) from the curated database of Varshney *et al.* [1] which is freely available through the Wormatlas: Altun, Z. F., Hall, D. H. (2002-2006) Wormatlas [2]. Available: `http://www.wormatlas.org`. Varshney *et al.* report a wiring diagram based on the original data from White *et al.* [3] augmented to include new serial section electron microscopy reconstructions. The connectome is composed of gap junctions which provide direct electrical couplings between neurons and therefore represent undirected (bidirectional) links between neurons. It is also composed of chemical synapses which use neurotransmitters to transmit signals at the synaptic cleft from a neuron to a target neuron and are therefore represented by directed links in the circuits. Here we consider the circuits of interneurons and motor neurons involved in two locomotion functions: forward and backward locomotion. The interneurons connect to motor neurons of classes A and B that control body wall muscles [3–5]. All neurons studied here are cholinergic and excitatory (ACh) except for RIM which uses neurotransmitter Glutamate and Tyramine and AIB which is glutamatergic (see Supplementary Note 6). The different types of synaptic interactions respect the symmetries found in the circuits.

Supplementary Note 2 - Network symmetry group

A network is a set of nodes $V = \{1, \ldots, N\}$ endowed with a connectivity structure defined by a set of edges E between pair of nodes. An edge $i \to j$ is interpreted as an arrow directed from node i to node j, which are said to be connected (or adjacent) to one another. The connectivity structure defined by the edge-set E can be put into the $N \times N$ adjacency matrix A, which has nonzero entries $A_{ij} \neq 0$ only if there is an edge $i \to j \in E$ connecting nodes i to j, and $A_{ij} = 0$ otherwise. We consider a weighted adjacency matrix to take into account the number of synaptic connections as given by [1].

The concept of permutation is as follows. A permutation of a network, denoted as P, is a bijective map $P : V \to V$ which pairs each node $i \in V$ with exactly one node $P(i) \in V$, and there are no unpaired nodes (whence the term bijective map). As a consequence, any permutation P has always a well-defined inverse, denoted as P^{-1}. Moreover, since permutations are orthogonal transformation, we have that $P^{-1} = P^T$, where P^T denotes the matrix transpose. Two permutations P_1 and P_2 can be composed (or multiplied), the result being another permutation. Composition of two permutations is written as $P_1 \circ P_2$, and the operation denoted by $\circ$ is called *composition law.* In the following, we omit for simplicity the symbol $\circ$ and write the composition as $P_1 \circ P_2 \equiv P_1 P_2$.

A set of permutations $\mathbf{G} = \{P_1, \ldots, P_n\}$ is said to form a **permutation group** under composition of its elements if it obeys the group axioms [6] listed below. **Definition of Permutation Group**:

1. existence of the **identity** $I \in \mathbf{G}$, defined as $I(i) = i$ for all i.
2. **associativity** of the composition law : $P_i(P_j P_k) = (P_i P_j)P_k$;
3. **closure** of the composition law: $P_i P_j \in \mathbf{G}$;
4. existence of the **inverse** P_i^{-1} for all $P_i \in \mathbf{G}$, defined by $P_i^{-1} P_i = P_i P_i^{-1} = I$.

In a network of size N there are $N!$ different ways to permute its nodes. The set of these $N!$ permutations obeys the group axioms listed above, so it forms a group. However, this is not the symmetry group of the network, because not all permutations are, in general, symmetries. To qualify as a network symmetry, P must preserve the connectivity structure, i.e., the network adjacency matrix A [6–9]. In other words, the permuted adjacency matrix PAP^{-1} must be identical to the original one: $A = PAP^{-1}$ if P is a permutation symmetry. Invariance of A under P is formally equivalent to the requirement that P commutes with A, so we have the formal definition of symmetry:

$$[P, A] \equiv PA - AP = 0 \quad \Longleftrightarrow \quad P \text{ is a permutation symmetry} . \tag{1}$$

Permutations which obey Eq. (1) are formally called network automorphisms [6]. In short, network symmetry and automorphism are synonyms of one another. For example, consider the circuit shown in Supplementary Fig. 1a, and the permutation P acting on it represented

by the matrix

$$P = \begin{array}{r} \text{AVBL} \\ \text{AVBR} \\ \text{RIBL} \\ \text{RIBR} \end{array} \begin{pmatrix} 0 & 0 & 1 & 0 \\ 0 & 0 & 0 & 1 \\ 1 & 0 & 0 & 0 \\ 0 & 1 & 0 & 0 \end{pmatrix} , \tag{2}$$

which swaps AVBL with RIBL, and AVBR with RIBR. This permutation is an automorphism, because the circuits before and after the action of P are exactly the same, as seen in Supplementary Fig. 1a. Moreover, it is easy to check that $[P, A] = 0$. Next, consider the action of the permutation Q shown in Supplementary Fig. 1b, given by the matrix

$$Q = \begin{array}{r} \text{AVBL} \\ \text{AVBR} \\ \text{RIBL} \\ \text{RIBR} \end{array} \begin{pmatrix} 0 & 0 & 0 & 1 \\ 0 & 1 & 0 & 0 \\ 0 & 0 & 1 & 0 \\ 1 & 0 & 0 & 0 \end{pmatrix} , \tag{3}$$

which exchanges AVBL with RIBR and leaves the other neurons fixed. Permutation Q is not an automorphism, because it does not preserve the connectivity between neurons. Indeed, before the action of Q, AVBL and AVBR are connected by a link with a weight=3, while after they are connected by a link with a weight=1. Thus, Q is not a symmetry, because it alters the connectivity structure of the circuit by changing the weights on the links. Consistently, we also have that $[Q, A] \neq 0$.

The set of all network automorphisms obeys all group axioms, so it forms a group. This group, denoted as $\mathbf{G}_{\text{sym}}(A)$, is called the **permutation symmetry group of the network** [6], and formally defined as:

$$\mathbf{G}_{\text{sym}}(A) = \{P \ : \ [P, A] = 0\} \ . \tag{4}$$

An algorithm to find perfect automorphisms of a given network is call Nauty, and it is given in Ref. [10], which is based on the well-known problem of testing isomorphism of graphs.

Supplementary Note 3 - Pseudosymmetries

A 25% variation across animals has been found in the connectivity of connectomes [1, 11]. For this reason, exact symmetries (= automorphisms) of the connectome are a simplification and an idealization. However, they should not be regarded as a falsification of symmetry

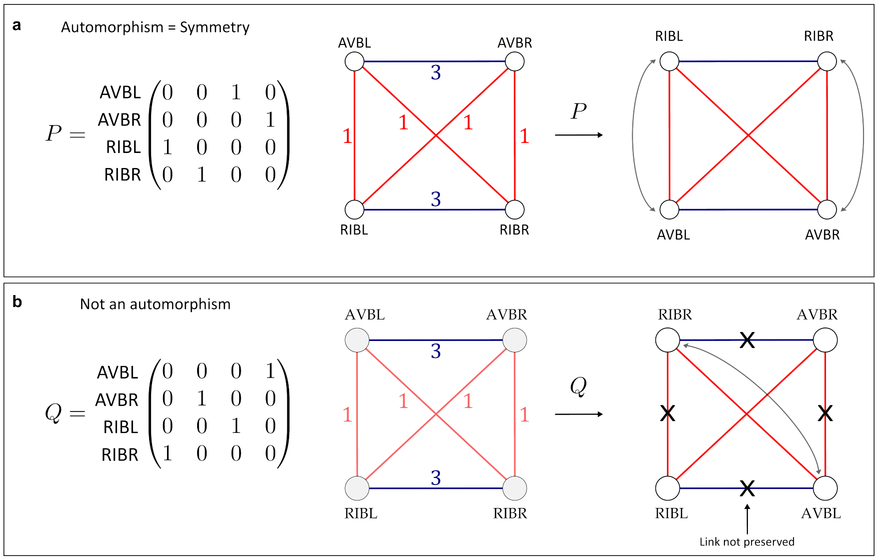

Supplementary Figure 1. **Symmetric and non-symmetric permutation**. **(a)** Permutation P Eq. (2) is a symmetry of the network preserving the connectivity of neurons (automorphism), and commutes with A: $[P, A] = 0$. **(b)** Permutation Q defined in Eq. (3) is not a symmetry of the network, because it changes the network connectivity by altering the weights of the links, so it does not commute with A: $[Q, A] = 0$.

principles, but rather as an intrinsic property of biological diversity. Symmetry principles, in biology, are invariably idealized and approximate: living systems do have to be sufficiently non-symmetric to evolve and diversify. Were it not so, the nature of exact symmetries would forbid any change in organisms' structure and functions. Furthermore, the animal displays a range of behaviors that are plastic and can change through learning and memory [12].

Unlike automorphisms, which are canonically defined by Eq. (1), the definition of pseudosymmetry depends on an additional parameter, a small number $\varepsilon > 0$, which, for our purposes, represents the 25% variation existing across animals.

A permutation P_ε is called a pseudosymmetry if the commutator $[P_\varepsilon, A]$ is non-zero but small

$$||[P_\varepsilon, A]|| \; = \; \varepsilon \tag{5}$$

that is, P_ε approximates an exact symmetry in the limit $\varepsilon \to 0$.

The norm of the commutator in Eq. (5), defined as

$$\Delta(P_\varepsilon) \; = \; ||[P_\varepsilon, A]|| \; \equiv \; \sum_{i \geq j} |A_{ij} - A_{P(i)P(j)}| \; , \tag{6}$$

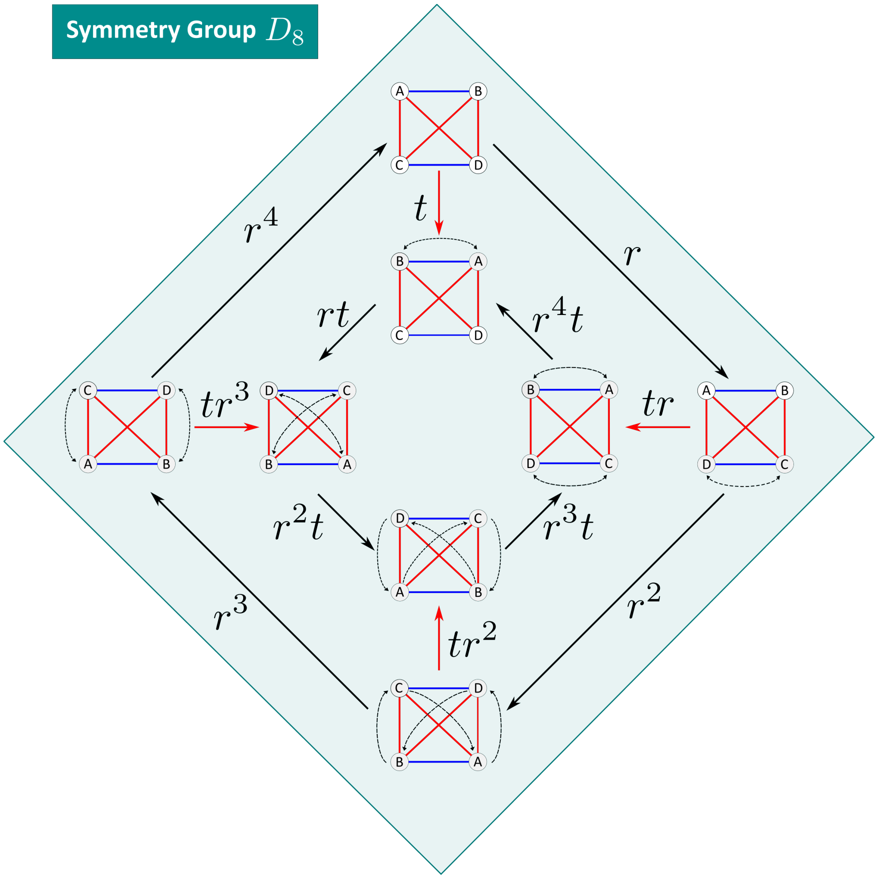

Supplementary Figure 2. **Dihedral symmetry group $\mathbf{D_8}$ of the forward gap-junction circuit (interneurons only).** The automorphisms r and t are the generators of this group, as shown. The structure of this group is then converted into the system of imprimitivity when this interneuron circuit is incorporated into the whole connectome. This is a general property of all functional circuits in the connectome, to be elaborated in a follow up paper.

counts the number of links where P_ε and A do not commute. The ideal limit of classical symmetry corresponds to $\Delta(P_\varepsilon) \to 0$, and we recover exact automorphisms. In general, the quantity $\Delta(P_\varepsilon) \to 0$ in Eq. (6) quantifies the deviation of P_ε from an ideal automorphism. Thus, we are lead naturally to the following definition of pseudosymmetry.

Definition of network pseudosymmetry– A permutation P_ε is called pseudosymmetry of the network if its deviation $\Delta(P_\varepsilon)$ from ideal automorphism is smaller than a given indetermination constant ε, i.e., $\Delta(P_\varepsilon) < \varepsilon M$, where M is the total number of links including the weights. In other words, we require pseudosymmetries to preserve at least a fraction $(1 - \varepsilon)$ of the total number of links.

Algorithm to find pseudosymmetries

In the present work, we choose the indetermination constant to be smaller than $\varepsilon < 0.25$, which represents the 25% variation in the connectivity of connectomes across animals [1, 3, 11, 13], as a condition for the permutation to be considered a pseudosymmetry. We then obtain the set of pseudosymmetries shown in the real circuits in the main text. Finding pseudosymmetries is relatively simple when the size of the network is small, because they can be determined by an exhaustive search as those permutations satisfying $\Delta(P_\varepsilon) < M\varepsilon$. To find the pseudosymmetries we compute for each permutation P the norm $\Delta(P_\varepsilon)$ given by Eq. (6), and we select only those such that $\Delta(P_\varepsilon) < M\varepsilon$. All pseudosymmetries found in the locomotion circuits represents transformation with indetermination constant ε below 25%. The list of the indetermination constants of all subgroups appears in Table I. We notice that pseudosymmetries of locomotion circuits are, in general, highly degenerate, and their number increases as a function of ε. Due to the fact that ε is relatively small, these real circuits can then be easily symmetrized to obtain the circuits with ideal symmetries with $\varepsilon = 0$. This is so, since the pseudosymmetries are relatively close to a perfectly symmetric circuit. The provided ideal circuits are examples of idealized symmetrical circuit and represents the closest ideal structure to the real one and at the same time respect the same symmetries as the pseudosymmetries of the real circuit. The real circuits (and only them) and their pseudo-symmetries remain the actual circuits to be studied. When the size N of the network is larger than $N > 20$, finding pseudosymmetries by using an exhaustive search becomes computationally impossible. In this case, pseudosymmetries should be determined as the solutions of a constrained quadratic assignment problem, to be elaborated and described in detail in a follow up paper.

Supplementary Note 4 - Factorization of the symmetry group

Factorization of the symmetry group into simple and normal subgroups is the fundamental tool for understanding the main results of this work. Descending to subgroups gives us useful information about the fine structure of the connectome, and eventually will allow us to identify its basic building blocks. Next, we explain the notion of subgroups and then the procedure to find the building blocks of the connectome through the factorization of its symmetry group. All definitions are standard in the group theory literature and appear in

Ref. [6].

Definition of Subgroup– A subset $\mathbf{H}$ of permutations selected from a group $\mathbf{G}$ is said to be a subgroup of $\mathbf{G}$ if the subset $\mathbf{H}$ forms itself a group (under the same composition law that was used in $\mathbf{G}$). The concept of subgroup is fundamental in mathematics and physics since it gives the structure of fundamental forces and particles [14].

Definition of Simple Subgroup– A simple subgroup is a nontrivial group whose only subgroups are the trivial group and the group itself. A group that is not simple can be broken into two smaller groups, a normal subgroup and the quotient group, and the process can be repeated, as explained next.

Definition of Normal Subgroup– Among all subgroups of a symmetry group, the normal subgroups, Fig. 1e, are particularly significant in this work, since they allow us to define the building blocks of the connectome. A subgroup $\mathbf{H}$ is said to be normal in a group $\mathbf{G}$ if and only if $\mathbf{H}$ commutes with every element $g \in \mathbf{G}$, i.e., $[g, \mathbf{H}] = g\mathbf{H} - \mathbf{H}g = 0$ (notice that the requirement is that $\mathbf{H}$ commutes with every g as a whole subgroup, not element by element).

More precisely, consider a group $\mathbf{G}$ and a subgroup $\mathbf{H} \leq \mathbf{G}$. For a given element $g \in \mathbf{G}$ we can form the set $\{gh : h \in \mathbf{H}\}$, which is called the **left coset** of $\mathbf{H}$ in $\mathbf{G}$. Thus we can use $\mathbf{H}$ to generate the collection of non-overlapping cosets $\mathbf{H}$, $g_1\mathbf{H}$, $g_2\mathbf{H}, \ldots.$ Note that while $\mathbf{H}$ is a subgroup, the cosets are, in general, simply sets. The crux of the matter is that if the cosets form themselves a group, then $\mathbf{H}$ is called a normal subgroup. Viceversa, if $\mathbf{H}$ is a normal subgroup, then the cosets do form a group, called the coset group. Next we explain which properties $\mathbf{H}$ must have in order to be a normal subgroup, or equivalently, for the cosets to form a group. Let $\mathbf{H}$ be a subgroup dividing $\mathbf{G}$ in N_c non-overlapping cosets. Since $\mathbf{G}$ may be, in general, a non-abelian group, the left cosets may differ from right cosets.

To be definite, in the following we consider only left cosets. Each left coset is of the form $g\mathbf{H}$ for some $g \in \mathbf{G}$. Let us consider two cosets $g_1\mathbf{H}$ and $g_2\mathbf{H}$. Since $\mathbf{H}$ is a subgroup, it must contain the identity element e, i.e. $e \in \mathbf{H}$. Therefore $g_1e = g_1$ is in the coset $g_1\mathbf{H}$. Analogously, $g_2e = g_2$ is in the coset $g_2\mathbf{H}$. Now, if cosets behave like a group, then the product g_1g_2 must be in the product of two cosets, that is $g_1g_2 \in (g_1\mathbf{H})(g_2\mathbf{H})$. Since g_1g_2 is also in the coset $g_1g_2\mathbf{H}$, then the product of any element in the first coset with any element in the second coset should be in the coset $g_1g_2\mathbf{H}$, i.e., $(g_1\mathbf{H})(g_2\mathbf{H}) = g_1g_2\mathbf{H}$. To see when this happens, consider an arbitrary element in the first coset $g_1\mathbf{H}$ and call it g_1h_1, and an

element in the second coset g_2h_2. Multiplying these two elements we get $g_1h_1g_2h_2$. If this is in the coset $g_1g_2\mathbf{H}$, then this product must be equal to $g_1g_2h_3$ for some h_3. Starting from this equation we can write:

$$\begin{aligned} g_1h_1g_2h_2 &= g_1g_2h_3 \\ h_1g_2h_2 &= g_2h_3 \\ g_2^{-1}h_1g_2h_2 &= h_3 \\ g_2^{-1}h_1g_2 &= h_3h_2^{-1} \ . \end{aligned} \tag{7}$$

Since $\mathbf{H}$ is a subgroup, the right hand side of Eq. (7) is in $\mathbf{H}$, i.e. $h_3h_2^{-1} \in \mathbf{H}$. As a consequence, also $g_2^{-1}h_1g_2$ is an element of $\mathbf{H}$, so we have in general that $g_2^{-1}\mathbf{H}g_2 \in \mathbf{H}$. In a similar way, one can prove that $\mathbf{H} \in g_2^{-1}\mathbf{H}g_2$, and thus conclude that

$$g_2^{-1}\mathbf{H}g_2 = \mathbf{H} \quad \rightarrow \quad [g_2, \mathbf{H}] = 0 \ . \tag{8}$$

To recap, we just proved that if $\mathbf{H} \leq \mathbf{G}$ is a subgroup and the cosets form a group, then it must hold true that $[g, \mathbf{H}] = 0$ for any $g \in \mathbf{G}$. In a similar way it can be proven that the converse is also true, that is, if $[g, \mathbf{H}] = 0$ then the cosets form a group. If this happens, then $\mathbf{H}$ is called a **normal** subgroup, denoted as $\mathbf{H} \trianglelefteq \mathbf{G}$, and the coset group is called **quotient** subgroup, denoted as $\mathbf{G}/\mathbf{H}$. Every group $\mathbf{G}$ has at least two normal subgroups, which are the identity $\{e\}$ and the group itself $\mathbf{G}$. If these are the only normal subgroups then $\mathbf{G}$ is called a **simple group**. In other words, a simple group does not have any quotient subgroups, and for this reason simple groups represent the building blocks of other groups. Normal subgroups (and only normal subgroups) can be used to decompose the symmetry group as a direct product, as we discuss next.

Definition of Direct Product Factorization– To explain the factorization of a group as a direct product of normal subgroups, it is useful to introduce the following notation. Let us consider a permutation group $\mathbf{G}$ and suppose that K is a subset of G. Then, we define the **support** of K by:

$$\text{supp}(K) = \{i \in V \mid P(i) \neq i \text{ for at least one } P \in K\} \ . \tag{9}$$

Then, suppose that two subsets $\mathbf{K}$ and $\mathbf{H}$ of a group $\mathbf{G}$ have non-overlapping supports, that is $\text{Supp}(K) \cap \text{Supp}(H) = \emptyset$, then all elements in $\mathbf{K}$ commute with those in H, i.e., $[K, H] = 0$. Assume now that a group $\mathbf{G}$ can be partitioned into a collection of subsets $\{\mathbf{H_1}, \mathbf{H_2}, \times, \mathbf{H_n}\}$ such that for any pair $\mathbf{H_i}$ and $\mathbf{H_j}$, $i \neq j$, $\text{Supp}(\mathbf{H_i}) \cap \text{Supp}(\mathbf{H_j}) = \emptyset$. Also, assume that each

subset H_i cannot be further partitioned into smaller subsets with non-intersecting supports. The important point is that the subsets $\mathbf{H_i}$ found in this way are, by simple construction, the uniquely defined normal subgroups that factorize $\mathbf{G}$ into a direct product as:

$$\mathbf{G} \;=\; \mathbf{H_1} \times \mathbf{H_2} \times \ldots \mathbf{H_n} \; . \tag{10}$$

More concretely, take the sector of blue motor neurons in Fig. 4a (VB3, VB4, VB5, VB10, VB11, DB2, DB4, DB6, DB7, DB8) and its associated subgroup $\mathbf{S_{10}}$ and the subgroup $\mathbb{T}^{\rm ch}_{\rm F}$ which acts on the sector of touch neurons colored green PVCL and PVCR. If we apply any permutation of $\mathbf{S_{10}}$ to the blue motor neurons, then the neurons PVCL and PVCR in the other sector are not affected. For instance, a permutation of VB3 and VB4 is a symmetry that does not affect for instance the touch sector of interneuron PVCL and PVCR. This factorization is because VB3 and VB4 are both connected to PVCL and PVCR, and this is a strong constraint on the connections. Imagine now that we loss two of the links and VB3 connects only to PVCL and VB4 only to PVCR. The resulting circuit would still be symmetric since we can still permute VB3 with VB4. But to keep the symmetry of the whole network, this permutation now triggers the permutation of PVCL and PVCR. Thus, VB3 and VB4 would belong to the touch sector together PVCL and PVCR. We see how the subgroup structure imposes hard constraints in the network connectivity. The fact that the connectivity of the network is precisely structured to create subgroups which can be factorized is an interesting result since not all groups possess this property. Furthermore the factors are aligned with different broad classification of functions. This is an indication that these subgroups have biological significance. Thus, the subgroup structure suggests the segregation of neurons in the network according to function yet allowing integration since the neurons are connected in the same circuit.

In Supplementary Note 5 we will show that both forward and backward circuits, either of gap-junctions or chemical synapses, have symmetry groups which factorize as a direct product of normal subgroups that correspond to specific broad functional categories from the Wormatlas.

Supplementary Note 5 - Symmetry group of *C. elegans* locomotion circuit

Forward gap-junction circuit

The real circuit with the weights of the synapses is shown in Supplementary Fig. 3. The corresponding symmetry group is factorized as a direct product of 6 normal subgroups:

$$\mathbf{F}_{\rm gap} \;=\; [\mathbf{C_2} \times \mathbf{C_2}] \times [\mathbf{S_5} \times \mathbf{D_1} \times \mathbf{C_2} \times \mathbf{C_2}] \;. \tag{11}$$

The pair of subgroups $[\mathbf{C_2} \times \mathbf{C_2}]$ acts on the set of four interneurons (AVBL, AVBR, RIBL, RIBR), but does not move any motor neuron. For this reason, we put them together to form the composite subgroup $\mathbb{C}_{\mathbf{F_{gap}}}$, which we call **command subgroup of the forward gap-junction circuit** and define as:

$$\mathbb{C}_{\mathbf{F_{gap}}} \;=\; \mathbf{C_2} \times \mathbf{C_2} \;. \tag{12}$$

Similarly, the product $[\mathbf{S_5} \times \mathbf{D_1} \times \mathbf{C_2} \times \mathbf{C_2}]$ in Eq. (11) acts only on the motor neurons VB and DB, but not on the interneurons. Therefore, we put them together to form the composite $\mathbb{M}_{\mathbf{F_{gap}}}$, and we call it the **motor subgroup of the forward gap-junction circuit**, defined as

$$\mathbb{M}_{\rm F_{gap}} \;=\; [\mathbf{S_5} \times \mathbf{D_1} \times \mathbf{C_2} \times \mathbf{C_2}] \;. \tag{13}$$

The formal decomposition of the circuit into the functional categories is:

$$\mathbf{F_{gap}} \;=\; \mathbb{C}_{\mathbf{F_{gap}}} \times \mathbb{M}_{\mathbf{F_{gap}}} \;. \tag{14}$$

Backward gap-junction circuit

The real circuit is shown in Supplementary Fig. 4 with the weighted links. The symmetry group of the backward circuit of gap-junctions breaks into a direct product of command and motor normal subgroups as:

$$\mathbf{B_{gap}} \;=\; (\mathbf{C_2} \times \mathbf{C_2} \times \mathbf{C_2} \times \mathbf{C_2}) \times (\mathbf{S_{12}} \times \mathbf{D_6} \times \mathbf{C_2}) \;. \tag{15}$$

where the command subgroup is

$$\mathbb{C}_{\mathbf{B_{gap}}} \;=\; \mathbf{C_2} \times \mathbf{C_2} \times \mathbf{D_1} \;, \tag{16}$$

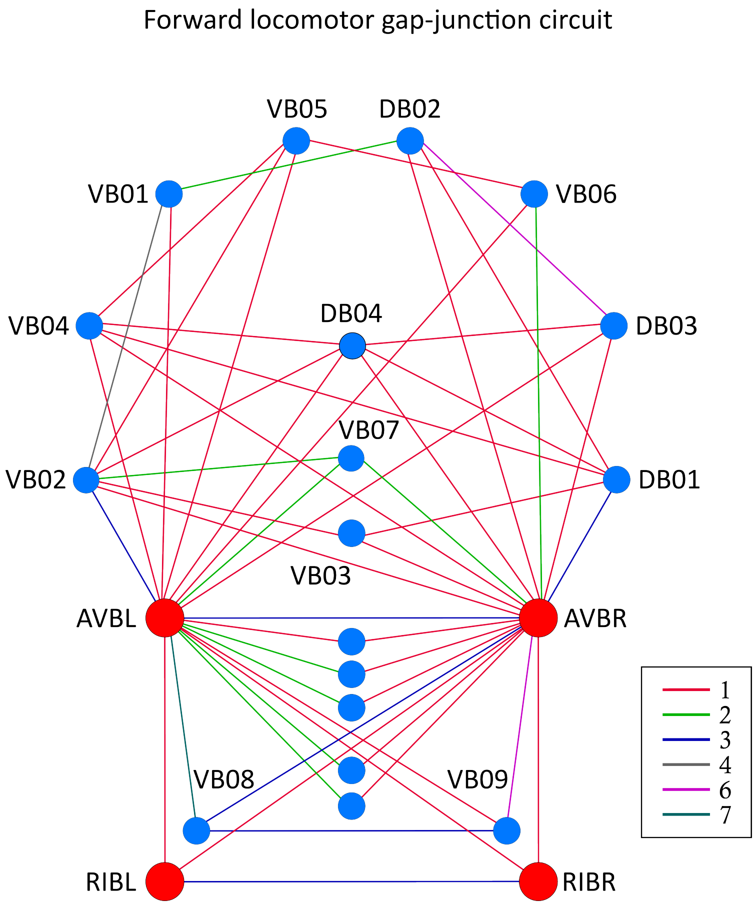

Supplementary Figure 3. **Real forward locomotion gap-junction circuit.** This circuit comprises 22 neurons divided in 2 sectors: the command-sector including the 4 interneurons (AVBL, AVBR, RIBL, RIBR); and the motor-sector including the remaining motor neurons.

acts on the command sector (AVAL, AVAR, AVEL, AVER, RIML, RIMR, AIBL, AIBR), and fix the motor sector, while the motor subgroup

$$\mathbb{M}_{\mathbf{B_{gap}}} = \mathbf{S_{12}} \times \mathbf{D_6} \times \mathbf{C_2}, \tag{17}$$

acts only on motor neurons DA and VA and leaves the interneurons fixed. The formal decomposition of the circuit is:

$$\mathbf{B_{gap}} = \mathbb{C}_{\mathbf{B_{gap}}} \times \mathbb{M}_{\mathbf{B_{gap}}} . \tag{18}$$

A Backward locomotor gap-junction circuit

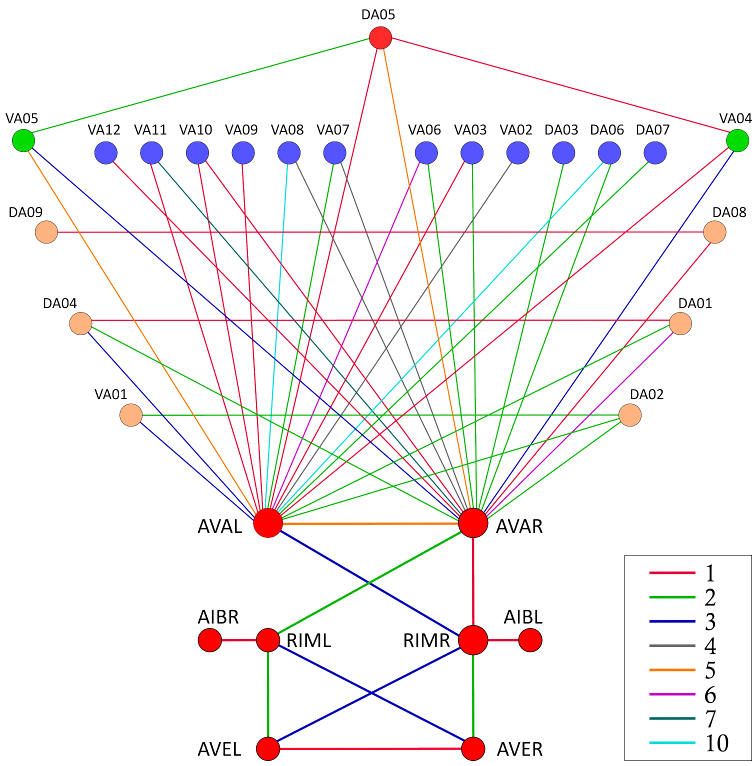

Supplementary Figure 4. **Real backward gap-junction circuit.** This circuit comprises 29 neurons connected by gap-junctions. These neurons form 2 disjoint sectors: the command-sector including 8 interneurons (AVAL, AVAR, RIML, RIMR, AIBL, AIBR, AVEL, AVER); and the motor sector formed by the remaining 21 motor neurons.

Forward chemical synapse circuit

We construct the forward circuit of chemical synapses using the same neurons of the forward gap-junction circuit discussed in Supplementary Note 5. In addition, we consider also the two neurons PVCL and PVCR, since they are connected to the other ones via chemical synapses (but not via gap-junctions). The resulting real circuit with the weighted links is displayed in Supplementary Fig. 5, and its pseudosymmetries are listed in Table I. We consider the different chemical synaptic connections according to the different neurotransmitters into excitatory and inhibitory. All neurons are cholinergic and excitatory (ACh) except for RIM which uses neurotransmitter Glutamate and Tyramine and AIB which is glutamatergic, as shown in Supplementary Table II. These different types of synaptic con-

nections do not affect the symmetries of the circuits and therefore we avoid to plot the type of neurotransmitter in the links of the chemical synapses circuits for clarity in all chemical circuits.

The corresponding (pseudo)symmetry group factorizes as the direct product of five normal subgroups in the following way:

$$\mathbf{F_{ch}} = (\mathbf{C_2}) \times (\mathbf{D_1}) \times (\mathbf{S_{10}} \times \mathbf{D_1}) \ , \tag{19}$$

The first subgroup $\mathbf{C_2}$ in Eq. (19) acts only on the pair of neurons (PVCL, PVCR) and leaves the rest fixed. For this reason, we name it **touch subgroup of forward chemical synapse circuit**, nd define as:

$$\mathbb{T}_{\mathbf{F_{ch}}} = \mathbf{C_2} \ , \quad \text{touch subgroup.} \tag{20}$$

The subgroup $\mathbf{D_1}$ acts only on the four interneurons, thus forming a composite subgroup named **command subgroup of the forward chemical synapse circuit**, which is defined as:

$$\mathbb{C}_{\mathbf{F_{ch}}} = \mathbf{D_1} \ , \quad \text{command subgroup.} \tag{21}$$

Lastly, the pair of subgroups $\mathbf{S_{10}} \times \mathbf{D_1}$ acts only on the motor neurons of this circuit, thus forming the **motor subgroup of the forward chemical synapse circuit**, which is defined by:

$$\mathbb{M}_{\mathbf{F_{ch}}} = [\mathbf{S_{10}} \times \mathbf{D_1}] \ , \quad \text{motor subgroup.} \tag{22}$$

The decomposition of this circuit is:

$$\mathbf{F_{ch}} = \mathbb{T}_{\mathbf{F_{ch}}} \times \mathbb{C}_{\mathbf{F_{ch}}} \times \mathbb{M}_{\mathbf{F_{ch}}} \ . \tag{23}$$

For simplicity we plot only the interneurons that connect to the motor neurons. Full circuit in Supplementary Fig. 6. All neurotransmitters are cholinergic and excitatory (ACh) except for RIM which uses neurotransmitter Glutamate and Tyramine and AIB which is glutamatergic (see Supplementary Note 6). These different types of synaptic interactions respect the symmetries of the circuits, see Supplementary Note 5.

Backward chemical synapse circuit

Since this circuit has a quite dense connectivity structure, for easier visualization, we plot it by separating two parts. Supplementary Fig. 6a shows the real circuit involved in the

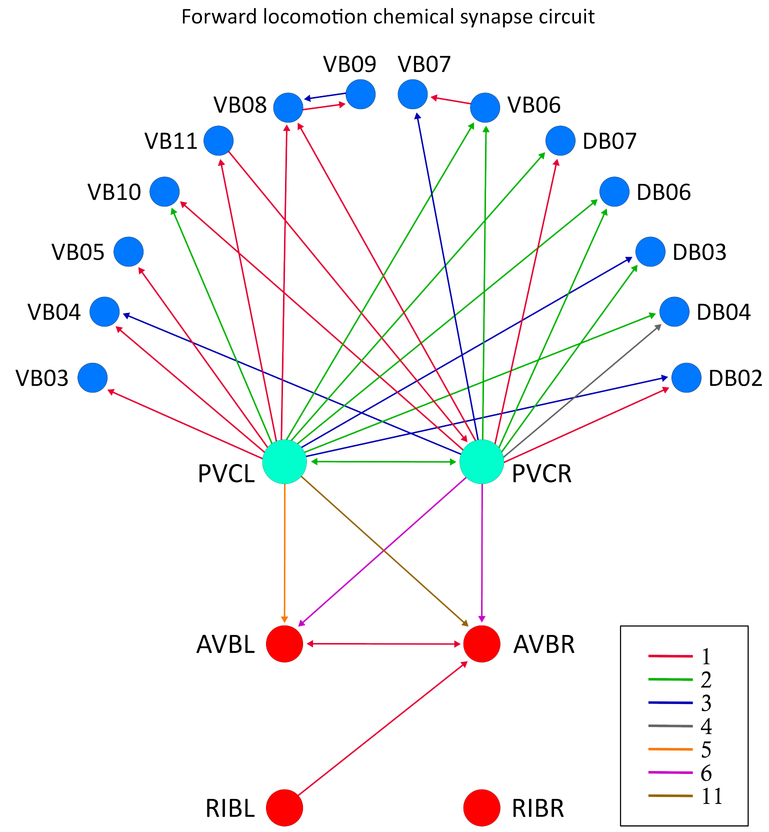

Supplementary Figure 5. **Real forward chemical synapse circuit.** This circuit comprises 20 neurons divided in 3 sectors: the touch-sector including the pair (PVCL, PVCR); the command-sector including the 4 interneurons (AVBL, AVBR, RIBL, RIBR); and the motor-sector including the remaining neurons. All neurons in this circuit are cholinergic.

touch-command subgroups. We then add the motor neurons in the class A and replot the interneurons involved in backward locomotion but only those that connect with the motor neurons in Supplementary Fig. 6b. These are the neurons AVA, AVE and AVD. Interneurons AIB and RIM in the command subgroup are not included for clarity of visualization because they do not contribute to the connections between the different sectors. We then obtain the real circuit displayed in Supplementary Fig. 6b involved in the touch-command-motor subgroups.

The symmetry group of the backward chemical synapse circuit shown in Fig. 4c is factorized as:

$$\mathrm{B_{ch}} \;=\; [\mathbf{C_2}] \times [\mathbf{C_2} \times \mathbf{C_2}] \times [\mathbf{S_5} \times \mathbf{S_4} \times \mathbf{S_3} \times \mathbf{C_2} \times \mathbf{D_1}]\ . \tag{24}$$

The touch sensitivity subgroup is composed of neurons AVD, the command interneuron

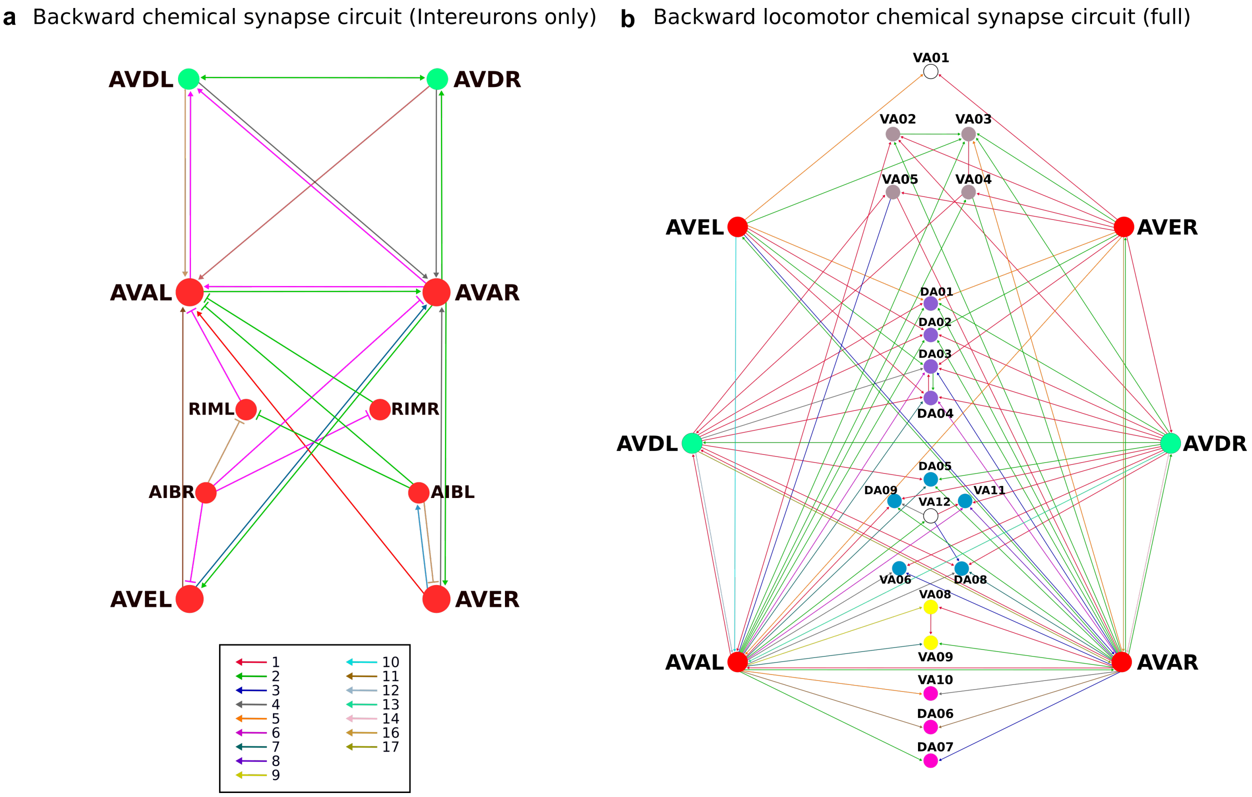

Supplementary Figure 6. **Real backward chemical synapse circuit.** **a**. We plot separately the interneurons for clarity. This part of the circuit comprises 10 neurons and the chemical synapse between them. These neurons form 2 disjoint sectors: the touch-sector including the pair (AVDL, AVDR); and the command-sector including the other 8 interneurons (AVAL, AVAR, RIML, RIMR, AIBL, AIBR, AVEL, AVER). All neurons in this circuit are cholinergic and excitatory (ACh), except for RIM and AIB which are inhibitory: RIM uses neurotransmitter Glutamate and Tyramine and AIB is glutamatergic. The inhibitory nature of their synaptic connections is shown graphically by T-headed arrows ($\dashv$, inhibitory links), as opposed to excitatory synapses represented by ordinary arrows ($\rightarrow$, excitatory links). The different types of synapses do not affect the pseudosymmetries of this circuit. **b**. We add the motor neurons to the circuit and plot only the interneurons that connect to the motor sector, for clarity. All neurons in this circuit are cholinergic.

subgroup of neurons AVA, AVE, AIB and RIM, and the motor subgroup consists of motor neurons VA and DA. The decomposition of this circuit is, respectively:

$$\mathbf{B}_{\text{ch}} = \mathbb{T}_{\mathbf{B_{ch}}} \times \mathbb{C}_{\mathbf{B_{ch}}} \times \mathbb{M}_{\mathbf{B_{ch}}} \ . \tag{25}$$

Supplementary Note 6 - Wormatlas functional categories on neurons

Broad functional categories of neurons are provided at the Wormatlas: `http://www.wormatlas.org/hermaphrodite/nervous/Neuroframeset.html`, Chapter 2.2 [2]. A classification for every neuron into four broad neuron categories is provided as follows: (1) *'motor neurons, which make synaptic contacts onto muscle cells'*, (2) *'sensory neurons'*, (3) *'interneurons, which receive incoming synapses from and send outgoing synapses onto other neurons'*, and (4) *polymodal neurons, which perform more than one of these functional modalities'*.

The Wormatlas classifies most neurons (some of them unknown) in further functional categories as well as provides the neurotransmitters. We reproduce the information from the Wormatlas used in the main text in Supplementary Table I and Supplementary Table II.

Forward circuit			
Neuron	Functional Category	Explanation	Neurotransmitter
AVB	interneuron	driver cell for forward locomotion	ACh
RIB	interneuron/motor polymodal	second layer interneuron, process of integration of information, locomotion	ACh
PVC	interneuron	command interneuron for forward locomotion, modulates response to harsh touch to tail	ACh
VB	motor (sensory)	locomotion (ventral), proprioception	ACh
DB	motor	forward locomotion (dorsal), proprioception	ACh

Supplementary Table I. Functional categories of the neurons in the forward circuit according to the Wormatlas.

Supplementary Note 7 - Blocks of imprimitivity

The correspondence of network building blocks and simple subgroups provides a rigorous theoretical characterization of the network connectivity structure and a natural interpretation of its broad functional categories according to the Wormatlas. However, a more accurate description of functionality should take into account also the splitting of these building blocks into finer topological structures. The fine structure corrections to the building blocks can be

Backward circuit			
Neuron	Function category	Explanation	Neurotransmitter
AVA	interneuron	command interneuron, locomotion, driver cell for backward locomotion	ACh
AVE	interneuron	command interneuron, drive backward movement	ACh
RIM	interneuron (motor)	second layer interneuron, process of integration of information, locomotion	Glu, Tyr
AIB	interneuron	first layer amphid interneuron, locomotion, food and odor-evoked behavior, lifespan, starvation response	Glu
AVD	interneuron	command interneuron, modulator for backward locomotion induced by head-touch	ACh
VA	motor	locomotion	ACh
DA	motor	backward locomotion	ACh

Supplementary Table II. Functional categories of the neurons in the backward circuit according to the Wormatlas.

obtained systematically through the concept of **system of imprimitivity** of a symmetry group **G**. All definitions appear in [6].

To define a system of imprimitivity we need first the notions of **transitivity** and **blocks**. A group **G** is said to be **transitive** on the set of nodes V if for every pair of nodes $i, j \in V$ there exist $P \in \mathbf{G}$ such that $P(i) = j$ (in other words, **G** has only one orbit). A group which is not transitive is called intransitive. A block is defined as a non-empty subset $\mathcal{B}$ of nodes such that for all permutations $P \in \mathbf{G}$ we have that:

- either P fixes $\mathcal{B}$: $P(\mathcal{B}) = \mathcal{B}$;
- or P moves $\mathcal{B}$ completely: $P(\mathcal{B}) \cap \mathcal{B} = \emptyset$.

If $\mathcal{B} = \{i\}$ or $\mathcal{B} = \{V\}$, then $\mathcal{B}$ is called a trivial block. Any other block is nontrivial. If **G** has a nontrivial block then it is called **imprimitive**, otherwise is called **primitive**.

The importance of blocks rests on the following fact. If $\mathcal{B}$ is a block for $\mathbf{G}$, then $P(\mathcal{B})$ is also a block for every $P \in \mathbf{G}$, and is called a conjugate block of $\mathcal{B}$. Suppose that $\mathbf{G}$ is transitive on the set of nodes V and define $\Sigma = \{P(\mathcal{B}) \mid P \in \mathbf{G}\}$ as set of all blocks conjugate to $\mathcal{B}$. Then the sets in Σ form a partition of the set of nodes V, and each element of Σ is a block for $\mathbf{G}$. We call Σ a **system of imprimitivity** for the (symmetry) group $\mathbf{G}$ [6].

In the text we have shown that the action of $\mathbf{G}$ on the system of imprimitivity Σ gives important information about the functionality of the neural circuits, provided $\mathcal{B}$ is not a trivial block.

Supplementary Note 8 - Circulant Matrices and Fast Fourier Transform

In this section we discuss the relationship between circulant matrices and discrete Fourier analysis (see Fig. 1g). In particular, we show that the eigenvalues of circulant matrices can be computed extremely fast through a routine of just $O(N \log N)$ operations, called Fast Fourier Transform (FFT).

We start the discussion by recalling that a circulant matrix $A = \mathrm{circ}(a_0, a_1, \ldots, a_{N-1})$ can always be written as a polynomial of the permutation matrix $P = \mathrm{circ}(0, 1, 0, \ldots, 0)$ of degree at most $N-1$, that is:

$$A = a_0 I + a_1 P + a_2 P^2 + ... + a_{N-1} P^{N-1} \ . \tag{26}$$

For instance, the low-pass filter:

$$\mathcal{L} = \mathrm{circ}(1,1) = \begin{bmatrix} 1 & 1 \\ 1 & 1 \end{bmatrix} , \tag{27}$$

can be written as $\mathcal{L} = I + P$. Next, we introduce the matrix F with entries F_{ab} defined as follows:

$$F_{ab} = \frac{1}{\sqrt{N}} \, e^{\frac{2\pi i}{N} ab} \ . \tag{28}$$

Matrix F is a unitary matrix ($F^{\dagger} = F^{-1}$) which represents the kernel of the discrete Fourier transform (DFT). Specifically, given a vector x, its DFT, denoted as $\tilde{x}$, is the vector defined as: $\tilde{x}_a = \sum_b F_{ab} v_b$. The crucial point is that the permutation matrix $P = \mathrm{circ}(0, 1, 0, \ldots, 0)$ is diagonalized by F, that is $P = F \Lambda F^{-1}$. This can be easily seen by calculating explicitly

the product $F^{-1}PF$, which reads:

$$(F^{-1}PF)_{ab} = \frac{1}{N}\sum_{k=0}^{N-1}\sum_{m=0}^{N-1} e^{-\frac{2\pi i}{N}ak} P_{km} e^{\frac{2\pi i}{N}mb} = \frac{e^{\frac{2\pi i}{N}b}}{N}\sum_{k=0}^{N-1} e^{\frac{2\pi i}{N}k(b-a)} = \delta_{ab}\ e^{\frac{2\pi i}{N}b}\ . \tag{29}$$

As a consequence of Eq. (29), any circulant matrix A is also diagonalized by F as

$$(F^{-1}AF)_{ab} = \sum_{n=0}^{N-1} a_n (F^{-1}P^nF)_{ab} = \delta_{ab}\sum_{n=0}^{N-1} a_n e^{\frac{2\pi i}{N}nb}\ , \tag{30}$$

so we can write down the eigenvalues $\{\lambda_a\}$ of A as

$$\lambda_a = \sum_{n=0}^{N-1} a_n e^{\frac{2\pi i}{N}na}\ , \quad a = 0, \ldots, N-1\ . \tag{31}$$

Eigenvalues $\{\lambda_a\}$ can be computed efficiently using the FFT of the vector $\vec{\alpha} \equiv \frac{1}{\sqrt{N}}(a_0, a_{N-1}, ..., a_1)^T$. To see this, we rewrite λ_a as

$$\begin{aligned}\lambda_a &= \sum_b (F^{-1}AF)_{ab} = \sum_{bk}(F^{-1}A)_{ak}F_{kb} = \sqrt{N}\sum_k (F^{-1}A)_{ak}\delta_{k0} \\ &= \sqrt{N}(F^{-1}A)_{a0} = \frac{1}{\sqrt{N}}\sum_b F_{ab}A_{b0}\ ,\end{aligned} \tag{32}$$

where we used the fact that F satisfies the following sum rules:

$$\begin{aligned}\sum_{b=0}^{N-1} F_{ab} &= \sqrt{N}\delta_{a0}\ , \\ \sum_{b=0}^{N-1} F^{-1}_{ab}F_{b0} &= \frac{1}{N}\delta_{a0}\ .\end{aligned} \tag{33}$$

Using the vectors $\vec{\alpha} \equiv \frac{1}{\sqrt{N}}(a_0, a_{N-1}, a_{N-2}, ..., a_1)^T$ and $\vec{\lambda} \equiv (\lambda_0, \lambda_1, ..., \lambda_{N-1})^T$, we can write Eq (32) in the simple form

$$F\vec{\alpha} = \vec{\lambda}\ , \tag{34}$$

which shows that the eigenvalues $\{\lambda_a\}$ of A are the components of the DTF of vector $\vec{\alpha}$. Since $F\vec{\alpha}$ can be evaluated in $O(N\log N)$ operations using a FFT, then the computational effort for diagonalizing a circulant matrix A requires $O(N\log N)$ operations, too. Thus, we can interpret the functionality of the circulant matrix as a fast way (almost linear in the number of nodes) to perform a Fourier Transform for processing of information.

[1] Varshney, L. R., Chen, B. L., Paniagua, E., Hall, D. H. & Chklovskii, D. B. Structural properties of the *Caenorhabditis elegans* neuronal network. *PLoS Comput. Biol.* **7**, e1001066 (2011).

[2] Altun, Z. F. & Hall, D. H. Wormatlas. (2002-2006). Available: `http://www.wormatlas.org`.

[3] White, J. G., Southgate, E., Thomson, J. N. & Brenner, S. The structure of the nervous system of the nematode *Caenorhabditis elegans*. *Philos. Trans. R. Soc. Lond. B* **314**, 1-340 (1986).

[4] Chalfie, M. *et al.* The neural circuit for touch sensitivity in *Caenorhabditis elegans*. *J. Neurosci.* **5**, 956-964 (1985).

[5] Zhen, M. & Samuel, A. D. T. *C. elegans* locomotion: small circuits, complex functions. *Cur. Opinion Neurobiol.* **33**, 117-126 (2015).

[6] Dixon, J. D. & Mortimer, B. *Permutation Groups*, Graduate Texts in Mathematics, 163 (Springer-Verlag, New York, 1996).

[7] Pecora, L. M. Sorrentino, F., Hagerstrom, A. M., Murphy, T. E. & Roy, R. Cluster synchronization and isolated desynchronization in complex networks with symmetries. *Nature Comm.* **5**, 4079 (2014).

[8] Sorrentino, F., Pecora, L. M., Hagerstrom, A. M., Murphy, T. E. & Roy, R. Complete characterization of the stability of cluster synchronization in complex dynamical networks. *Science Adv.* **2**, e1501737 (2016).

[9] Abrams, D. M., Pecora, L. M. & Motter, A. E. Focus issue: Patterns of network synchronization. *Chaos* **26**, 094601 (2016).

[10] McKay, B. D. Nauty user's guide (version 1.5), Tech. Rep. TR-CS-90-02, Australian National University (1990).

[11] Durbin, R. M. Studies on the development and organisation of the nervous system of *Caenorhabditis elegans*. [PhD thesis], University of Cambridge (1987).

[12] Giles, A. C., Rose, J. K. & Rankin, C. H. Investigations of learning and memory in *Caenorhabditis elegans*. Int. Rev. Neurobiol. **69,** 37-71 (2005).

[13] Hall, D. H. & Russell, R. L. The posterior nervous system of the nematode *Caenorhabditis elegans*: serial reconstruction of identified neurons and complete pattern of synaptic interactions.

J. Neurosci. **11**, 1-22 (1991).

[14] Weinberg, S. *The Quantum Theory of Fields* (Cambridge University Press, Cambridge, 2005).
